# Broadening the Scope of Binding Free Energy Calculations Using a Separated Topologies Approach

**DOI:** 10.1021/acs.jctc.3c00282

**Published:** 2023-07-24

**Authors:** Hannah M. Baumann, Eric Dybeck, Christopher L. McClendon, Frank C. Pickard, Vytautas Gapsys, Laura Pérez-Benito, David F. Hahn, Gary Tresadern, Alan M. Mathiowetz, David L. Mobley

**Affiliations:** † Department of Pharmaceutical Sciences, University of California, Irvine, Irvine, California 92697, United States; ‡ Pfizer Worldwide Research, Development, and Medical, 1 Portland Street, Cambridge, Massachusetts 02139, United States; § Computational Chemistry, Janssen Research & Development, Janssen Pharmaceutica N. V., Turnhoutseweg 30, B-2340 Beerse, Belgium; ∥ Department of Chemistry, University of California, Irvine, Irvine, California 92697, United States

## Abstract

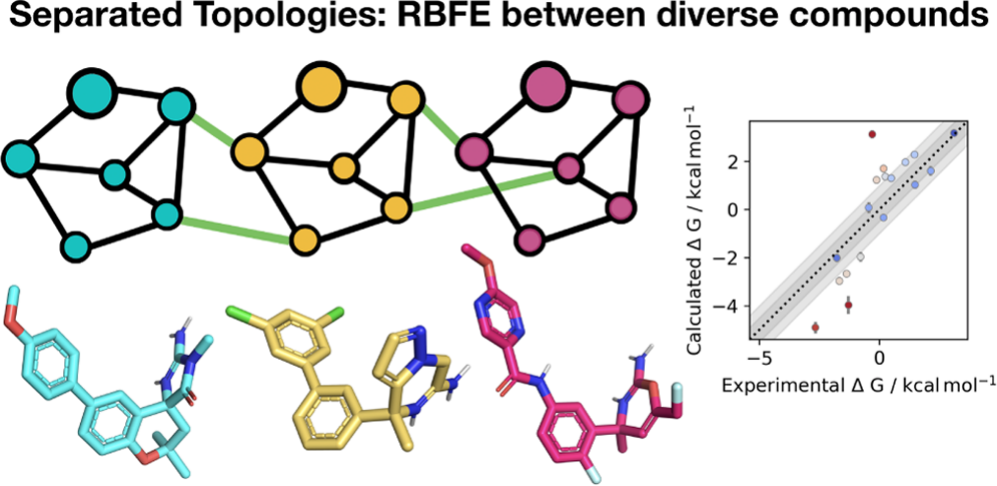

Binding free energy calculations predict the potency of compounds to protein binding sites in a physically rigorous manner and see broad application in prioritizing the synthesis of novel drug candidates. Relative binding free energy (RBFE) calculations have emerged as an industry-standard approach to achieve highly accurate rank-order predictions of the potency of related compounds; however, this approach requires that the ligands share a common scaffold and a common binding mode, restricting the methods’ domain of applicability. This is a critical limitation since complex modifications to the ligands, especially core hopping, are very common in drug design. Absolute binding free energy (ABFE) calculations are an alternate method that can be used for ligands that are not congeneric. However, ABFE suffers from a known problem of long convergence times due to the need to sample additional degrees of freedom within each system, such as sampling rearrangements necessary to open and close the binding site. Here, we report on an alternative method for RBFE, called Separated Topologies (SepTop), which overcomes the issues in both of the aforementioned methods by enabling large scaffold changes between ligands with a convergence time comparable to traditional RBFE. Instead of only mutating atoms that vary between two ligands, this approach performs two absolute free energy calculations at the same time in opposite directions, one for each ligand. Defining the two ligands independently allows the comparison of the binding of diverse ligands without the artificial constraints of identical poses or a suitable atom–atom mapping. This approach also avoids the need to sample the unbound state of the protein, making it more efficient than absolute binding free energy calculations. Here, we introduce an implementation of SepTop. We developed a general and efficient protocol for running SepTop, and we demonstrated the method on four diverse, pharmaceutically relevant systems. We report the performance of the method, as well as our practical insights into the strengths, weaknesses, and challenges of applying this method in an industrial drug design setting. We find that the accuracy of the approach is sufficiently high to rank order ligands with an accuracy comparable to traditional RBFE calculations while maintaining the additional flexibility of SepTop.

## 1 Introduction

Binding free energy calculations are a physically rigorous approach to prospectively estimate ligand potency, even before the ligand is synthesized. Although initial applications of these methods were reported decades ago,^1−3^ recent advances in computing technology, such as graphical processing units and low-cost parallel computing, have enabled the pharmaceutical industry to routinely and successfully apply these methods to drug discovery projects.^4−10^

First, we review two common computational methods to estimate binding free energy values: relative binding free energy and absolute binding free energy. Then, we introduce a third approach that combines the strengths of these two, giving accurate results and providing an alternative when neither absolute nor relative calculations are well suited to the problem. In this paper, we focus on alchemical approaches, which employ an unphysical path to connect two physical end states in order to obtain free energy differences.

The so-called “relative” binding free energy (RBFE) approach calculates the difference in potency between two similar ligands. During the simulations, one ligand is converted into the other by alchemical transformations of the atoms that vary between the two ligands. Common atoms from one ligand are mapped on top of those from the other ligand, resulting in either a single set of coordinates of the two ligands in the end states (single topology) or a single set of coordinates for the common core while representing atoms that differ between the two ligands separately (hybrid topology).^11^ The single and the hybrid topology approaches are based on having a common core, distinct from the Separated Topologies approach, as described below. The common core is often defined as the maximum common substructure between the two ligands^12,13^ (or between multiple ligands^14,15^); however, additional mapping ideas are possible.^16^ The RBFE approach is less computationally demanding and has lower statistical uncertainties than absolute binding free energy (ABFE) calculations. It also has advantages, e.g., if both ligands introduce similar conformational changes in the protein, such slow motions do not have to be sampled since the binding site is never empty. This RBFE approach is most suitable for comparing the binding of related ligands and is routinely applied in drug discovery.^4−7,17,18^

The RBFE approach, however, essentially requires that the two ligands share a common scaffold that can be preserved (allowing the ligand to retain its binding mode) while modifying atoms that are not retained. This requirement for a common scaffold provides a critical challenge, especially in early-stage drug discovery, where complex modifications to ligands are common. This limitation prevents these techniques from being as useful as they could be in guiding drug design. Scaffold hopping approaches^19,20^ allow for larger transformations, for example, ring opening and ring size change transformations; however, the transformation size is limited since ligands still need to share a common core. Moreover, in common practice, RBFE calculations need ligands to have a shared binding pose and/or protein conformation. Additionally, RBFE calculations require atom mapping, and the construction of the “dummy atoms” must be done carefully to ensure that the energy contribution of the decoupled dummy atoms cancels out between the complex and the solvent legs of the thermodynamic cycle.^21^ For example, if dummy atoms are connected to the rest of the system by more than one bond, the energy contribution does not cancel out automatically.^7^ Additionally, angle and torsional terms can introduce considerable complexity if not handled with great care.^21^ This concern does not apply to systems where all ligand atoms are transformed into dummy atoms, such as in ABFE.

Alternatively, the “absolute” approach computes the potency of individual ligands directly, usually through a thermodynamic cycle where a ligand is decoupled in the binding site—meaning all its nonbonded interactions are turned off—and coupled in the solvent where the interactions are turned back on.^22^ Since ligands are treated individually, they do not need to share a common scaffold and can be structurally diverse. This means that ABFE could be used even in early project stages where structurally diverse ligands are common and has been proposed to serve as a final scoring stage in virtual high throughput screening before selecting molecules for experimental testing.^9,23^ Recent studies showed that ABFE can achieve a good correlation between predicted and experimental binding free energies across different systems^24−27^ and can even be used to estimate binding to different proteins, allowing computation of the selectivity of ligands for a particular target.^28^

However, a major limitation of the ABFE approach is that it can produce larger statistical uncertainties in the predicted potency of the ligand compared to relative approaches (see below), especially in systems where the target undergoes larger conformational changes upon ligand binding. For example, consider a protein undergoing a slow flap-closing motion upon ligand binding, such as HIV protease;^29^ an ABFE calculation would need to sample the unbound state to correctly compute the true binding free energy. Such protein motions are not sampled on the typical timescale of molecular dynamics (MD) simulations, resulting in inaccurate potency predictions. Slow degrees of freedom require long sampling times or the use of enhanced sampling techniques, which can increase computational costs.^26^ As a result, the ABFE method is not routinely applied in drug discovery projects. If, when the ligand is decoupled, all structures are metastable in something like the bound state, one can obtain relative results from ABFE without having to sample apo–holo protein conformational transitions. However, this is not always the case, as discussed in Section 6

An alternate approach for RBFE, “Separated Topologies”, which we will refer to as “SepTop” throughout this paper, has the potential to combine the advantages of ABFE and standard RBFE. This protocol performs two ABFE calculations simultaneously in opposite directions by (alchemically) inserting one ligand into the binding site while removing the other ligand at the same time. In contrast to the standard RBFE protocol, the two ligand topologies are completely separate (meaning there is no common core), making atom mapping unnecessary. Consequently, the two ligands can be structurally diverse and do not need to share a fully overlapping binding mode and/or a common scaffold, overcoming the limitations of the common RBFE approach mentioned above. This protocol also never needs to sample the apo state of the protein as long as the protein retains its holo structure in the presence of both ligands since one ligand (or a fraction of both) is always present in the binding site. Therefore, larger protein conformational changes between the bound and unbound state never need to be sampled, giving this approach a benefit in comparison to the absolute protocol. Additionally, if both ligands have the same non-zero charge, the SepTop approach conserves that charge during the transformation in the binding site, while in ABFE, the net charge in the binding site changes, which can lead to a wide variety of sampling and theoretical problems.^30−32^

The SepTop method was introduced in 2013 in a proof-of-principle study.^33^ In that study, the authors compared three RBFE methods, a single topology approach, dual topology, and SepTop, and studied the binding of two ligands to an engineered site in cytochrome *c* peroxidase. In dual topology calculations, a separate set of coordinates is used for each ligand, in contrast to the setup in single or hybrid topology approaches. Separated Topologies can be considered a subcategory of dual topology where ligands are restrained spatially to a specific area. In the study by Rocklin et al.,^33^ dual topology referred to a different subcategory, the “linked dual topology approach” where the ligands are restrained to each other using, e.g., distance restraints.^11^ Rocklin et al. found that all three approaches gave comparable results when ligand reorientation was not required, while in the presence of multiple ligand binding modes, SepTop had advantages over the other RBFE approaches. In this latter case, only SepTop gave accurate results by treating individual poses separately using orientational restraints.^34^

In the prior SepTop work of Rocklin et al., the RBFE between binding modes was calculated, and the contributions of different poses were combined to obtain the overall free energy difference. This earlier study seemed to show that the approach is viable but did little to make it practical for applications or to show that it could be useful for pharmaceutically relevant systems. In addition to SepTop, there have been other recent reports of similar approaches to resolving the difficulties of ABFE calculations, such as dual topology approaches^35^ and the alchemical transfer method (ATM).^36^

In this paper, we reintroduce SepTop and show that it works on pharmaceutically relevant systems. We develop a prototype Python package to set up SepTop calculations in GROMACS^37^ and discuss heuristics for picking atoms for orientational restraints. We test the method on several diverse, pharmaceutically relevant systems and report performance and the resulting insights into strengths, weaknesses, and challenges. We first test the approach on systems with small ligand transformations, allowing us to compare SepTop to standard RBFE and validate that it yields correct binding free energies. On more ambitious transformations, we find that SepTop performs well, even when such transformations fall outside the scope of standard RBFE methods.

## 2 Methods

### 2.1 Thermodynamic Cycle for SepTop Computes RBFE by Running Two ABFE Calculations in Opposite Directions

The relative binding free energy between two ligands A and B, ΔΔ*G*_bind_, can be obtained by transforming one ligand into the other ligand both in the solvent and in the binding site.
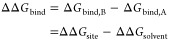
1

**Figure 1:**
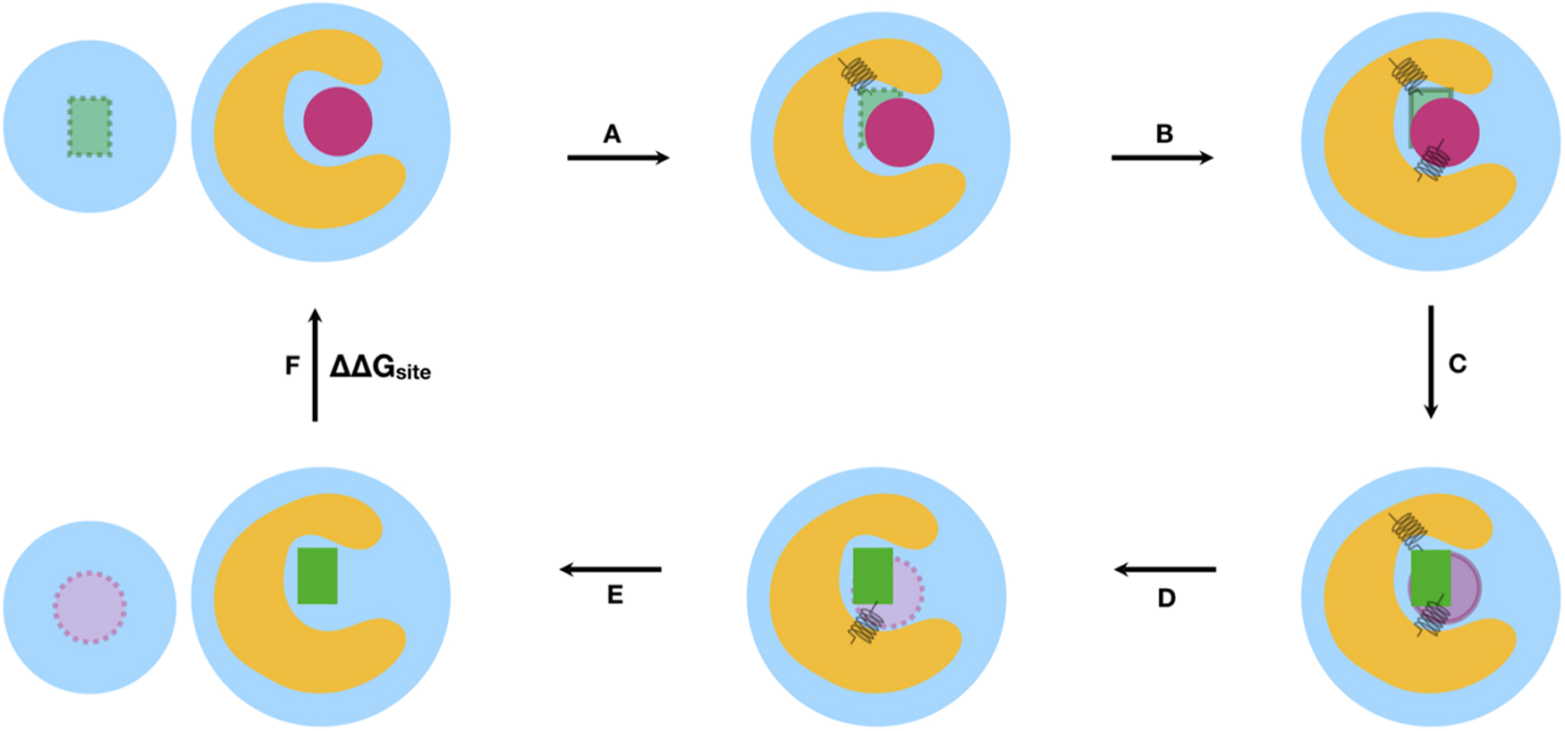
Thermodynamic cycle for SepTop for computing the free energy difference between two ligands in the binding site (F). The non-interacting dummy ligand (green) is inserted into the binding site and restrained using orientational (Boresch-style) restraints^34^ (A). The van der Waals (vdW) interactions of the green ligand are turned on, and the magenta ligand is restrained (B), and in the next step, the electrostatic interactions of the green ligand are turned on while at the same time, the electrostatics of the magenta ligand are turned off (C). Then, vdW interactions of the magenta ligand are turned off while at the same time releasing restraints on the green ligand (D). Lastly, the restraints of the now dummy magenta ligand are released analytically, and the ligand is transferred into the solvent (E). The free energy difference between the ligands in the solvent was obtained separately by running either two absolute hydration free energy calculations or a relative hydration free energy calculation using a SepTop approach.

In SepTop, we obtain the relative free energy difference between two ligands in the binding site by running what is essentially two ABFE calculations at once in opposite directions. The thermodynamic path for the transformation of one ligand into the other ligand in the binding site (Δ*G*_site_) is shown in Figure 1. To obtain the relative solvation free energy, Δ*G*_solvent_, we perform two absolute hydration free energy calculations if all ligands are neutral. If, on the other hand, ligands have the same non-zero charge, we use a SepTop protocol in the solvent leg in order to preserve the net charge in the system (see Section 4.3).

Restraints are required to keep the weakly coupled and fully decoupled ligand in the binding site region and thereby reduce the phase space that needs to be sampled. In this study, we apply orientational restraints, which we call “Boresch-style” restraints (after the seminal work of Boresch et al., which first employed these to make ABFE calculations practical).^34^ In principle, however, numerous other kinds of restraints could be used for this step (affecting only the efficiency), and an assessment of the convergence of different pose restraint strategies is outside the scope of the present study. The efficiency of the approach naturally depends on the choice of restraints, e.g., if two ligands share a similar shape, simulations would likely be most efficient if the shapes of the two ligands overlap well in all alchemical states. If the two ligands have a similar shape, one could restrain the shape of the first ligand to the shape of the second ligand so that in states where one of the ligands is only weakly interacting or fully decoupled, it samples the phase space of the interacting ligand it is restrained to. Such issues have not yet been carefully explored and are not the focus of the present work.

### 2.2 We Developed Heuristics for Automatically Picking Suitable Atoms for Boresch-Style Restraints

The orientational restraints used here restrain 3 atoms in the protein and 3 atoms in the ligand through 1 distance, 2 angle, and 3 dihedral restraints. Although the binding free energy should be independent of the atom selection,^34,38^ the selection can impact the convergence and (numerical) stability of the simulations. Therefore, we implemented a tool that selects suitable atoms for the restraints.

Multiple approaches to selecting stable atoms for Boresch-style restraints have been reported,^10,27,39,40^ with some selection criteria being similar across implementations while other criteria differ. In these studies, equilibration simulations ranging from 1^39^ to 20 ns^27^ were performed to help identify stationary points in the protein and ligand. Different approaches for identifying these stable structural elements were explored, such as selecting sets of atoms with the most frequent hydrogen bond and salt bridge interactions during MD,^39^ looking for buried residues with likely low mobility by calculating the minimal solvent-exposed surface area from the MD simulation^10^ or choosing a combination of protein and ligand atoms that result in the lowest standard deviation across all six bond, angle, and dihedral terms calculated across the equilibration simulation.^27^ All methods have in common that only protein backbone (and C β) atoms were considered for the restraints. Approaches varied in the selection of the ligand atoms. Neighboring heavy atoms were considered,^39^ while others only considered heavy atoms within rigid scaffolds^10^ to avoid restraining rotatable bonds and therefore locking-in conformations. A different approach selects ligand atoms with the farthest distance from one another.^40^

**Figure 2:**
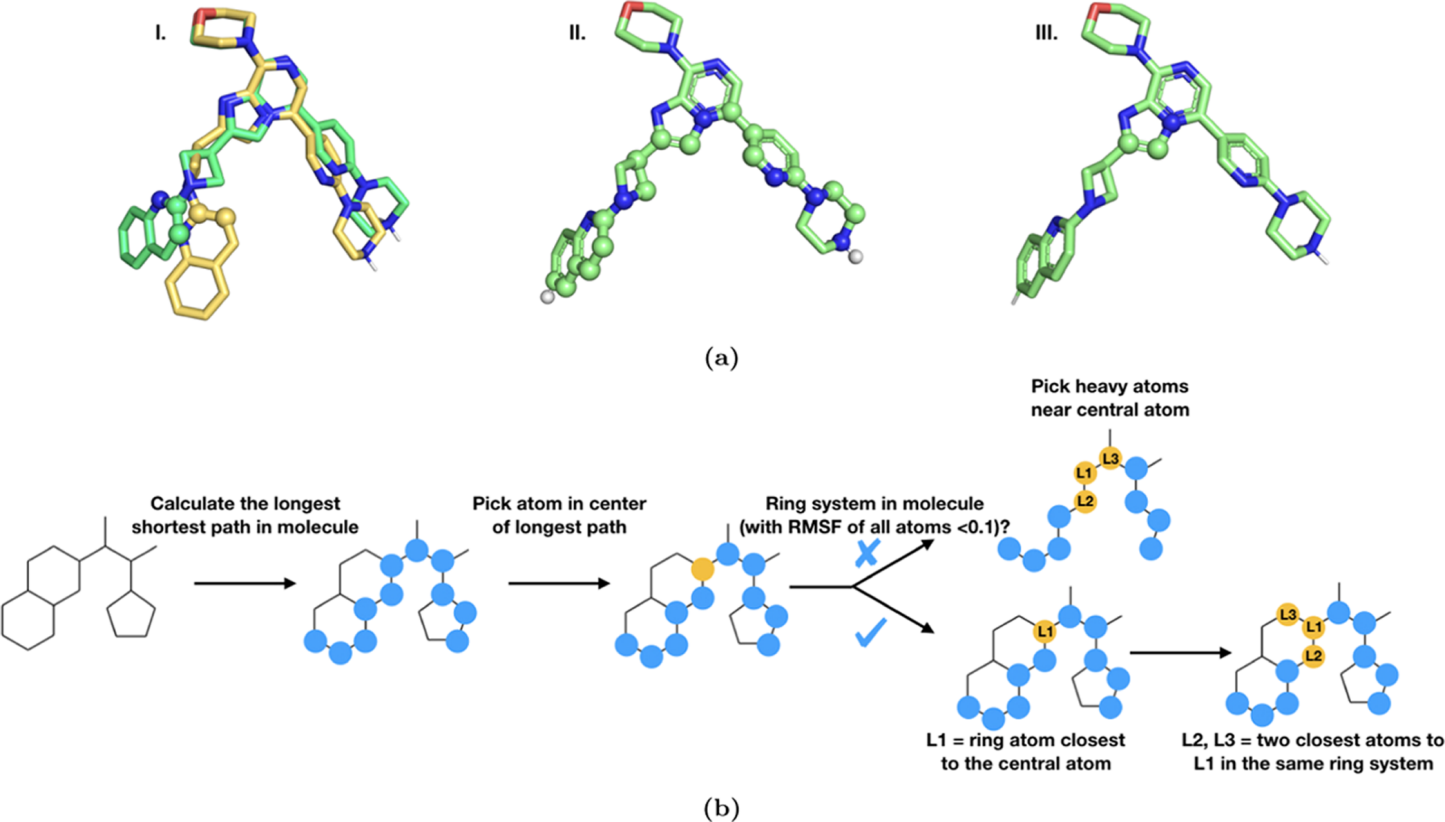
Choices made in selecting suitable ligand atoms for the restraints. (a) Selection of ligand atom for Boresch restraints for a PDE10 ligand. (I) Our initial version of the algorithm picked atoms from the largest ring system closest to the ligand center of mass (COM). The three selected atoms are shown in spheres. During simulations, this ring rotated from its initial structure (green) to a different binding mode (yellow). (II) The new algorithm calculates all shortest paths between two atoms in the molecule and selects the longest path among those (shown in spheres). (III) The three-ring atoms closest to the middle of the longest path are selected for the Boresch restraints. (b) Selection algorithm of ligand atoms based on the longest path in the molecule.

Our approach builds on previous work by developing a heuristic algorithm aimed at identifying atoms that are likely to remain relatively stable as long as the ligand maintains the same binding mode, thus allowing them to effectively restrain the ligand’s motion as ligand interactions are removed. An example is shown in Figure 2a. Restraining ligand atoms that change their position during the simulation (Figure 2a.I) may lead to slow convergence while restraining stable ligand atoms (Figure 2a.III) can be more efficient. Similarly, convergence can be negatively affected if protein atoms involved in the restraints change their position substantially during a simulation. The algorithm is therefore designed to pick protein atoms that are likely to maintain a fairly constant position. Our aim here is to develop a restraining protocol that should work well on a large fraction of systems. However, we are aware that it is possible that no single algorithm will be ideally suited to all cases. The code for this restraining protocol is detailed below and can be found in the GitHub repository SeparatedTopologies.^41^

As one option, the equilibrated and minimized complex structure could be used to determine the Boresch restraint atoms. However, this has the drawback that it may not always be obvious which atoms will be stable from a single set of coordinates. A way to get around this is to use an entire trajectory. For example, protein–ligand complexes are often equilibrated, and some data is collected prior to binding free energy calculations. Such simulations can be analyzed to help select atoms for restraints. In this work, we designed our restraints selection tool so that, if an input trajectory is provided, it is used to ensure that only atoms with relatively minimal fluctuations in their positions are considered as possible reference atoms for restraints. In particular, all protein and ligand atoms with a root mean square fluctuation (RMSF) > 0.1 Å are excluded and not considered for the restraints. While the ideal cutoff value might depend on the length of the input simulation, we found this cutoff at 0.1 Å to be a practical threshold for simulations of 2 ns where frames are saved every 4 ps.

For ligand atoms, reference atoms for restraints can be chosen either automatically or by the user. The latter can be very useful if a ligand series has a structural element that is known to be stable or to be involved in key interactions in the binding site. We implemented an option for users to define their selections through a substructure search via SMARTS patterns. Automatic selection of ligand atoms, on the other hand, can be used when no prior information on stable ligand groups is available or if the series does not share a common group. Here, the algorithm selects ligand atoms in a central ring system since a central ring system is likely more stable than other parts of the molecule.

More specifically, the tool computes all shortest paths between two atoms in the molecule graph, selects the longest path among those, and picks the ring atoms closest to the middle of the longest path (Figure 2b). The algorithm then picks the center of the molecule using the longest path instead of the center of mass (COM) because we found that in one of the systems (PDE10), the later method led to the selection of atoms in an outer ring system, which exhibited significant movements away from its original orientation during the simulation (Figure 2a.I). This suggested that if a distal ring system is used, even small local rearrangements of the ring could incorrectly appear to be substantially changing the ligand binding mode (as far as restraint calculations are concerned). In contrast, using a relatively central ring system to define restraints will ensure the detection of substantial changes in ligand binding mode (such as an overall rotation or translation of the ligand in the binding site) and will certainly result in substantial changes in the relevant degrees of freedom in this case. Here, the PDE10 system helped improve the restraining protocol and is included here because it helped us develop the heuristics employed in our selection algorithm; however, it is not a focus of our study as we moved to other systems as soon as the selection algorithm was in place and did not further study PDE10.

**Figure 3:**
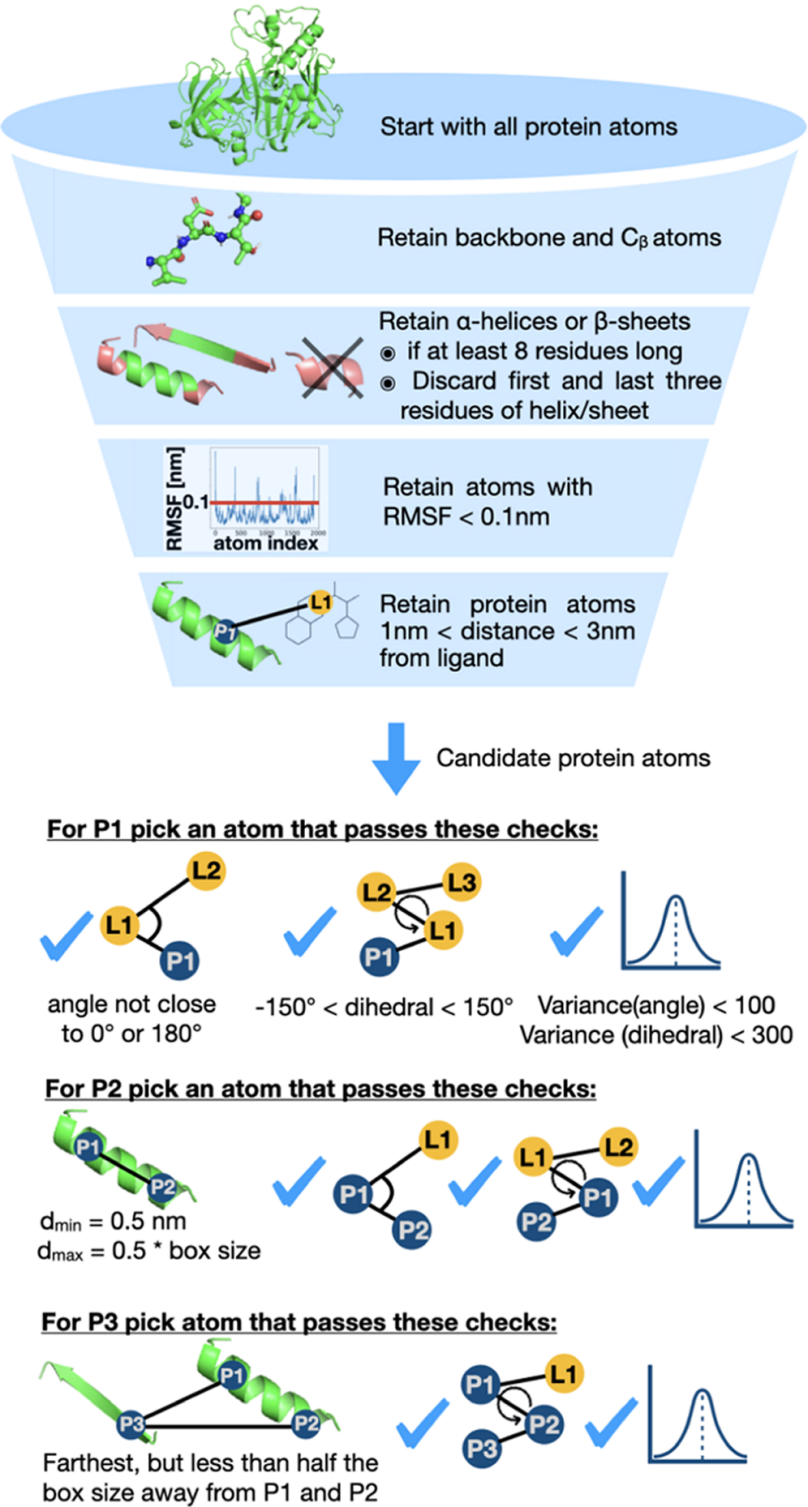
Selection of suitable protein atoms for the restraints based on finding atoms that are likely to remain at a constant position in MD simulations and other criteria that ensure the numerical stability of the simulations.

Boresch-style restraints also require the selection of three reference protein atoms; for these, our algorithm initially considers all protein atoms and then progressively filters out undesirable atoms (Figure 3). In this filtering process, the algorithm retains protein backbone atoms (as well as C-β atoms) that are in the middle of an α helix or β sheet since those are typically the most stable secondary structure elements. A trivial approach from here would be to select α helices only. However, this fails for proteins like galectin, which do not have any helices. Therefore, we find that a more rigorous approach is to include backbone atoms in α helices if those are the dominant secondary structure and, if not, include both backbone atoms from helices and β sheets. The algorithm picks atoms from central residues in those helices/sheets since outer residues can be more flexible and less stable. As mentioned above, only protein atoms with an RMSF < 0.1 Å are retained. In addition to this, atoms have to be at least 10 Å and no more than 30 Å away from the ligand. The rationale for the 10 Å minimum distance is that binding site residues can undergo conformational changes upon ligand binding and, therefore, might be less stable as an anchor point.

Adequate reference atoms in the protein must also satisfy several other factors. For example, if either of the two angle restraints is too close to 0 or 180°, the simulations crash due to numerical instability. To avoid this, we implemented criteria to avoid near 0 or 180° angle restraints, more specifically that the two angle cutoffs *a*_cut_ are above 10 *RT*
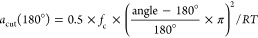
2
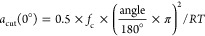
3where *f*_c_ is the force constant.

In addition, atoms involved in the restraints must be sufficiently near one another, often less than half the shortest box edge away, to avoid problems due to periodic boundary conditions and the minimum-image convention. While larger separations would not, in principle, be a problem, restraints in certain simulation packages like GROMACS do not smoothly handle the minimum-image convention. For example, in one system (estrogen receptor α), a small movement of atoms involved in the restraints led to one of the restrained dihedral angles jumping between being computed “through” vs “around” the box, based on the minimum-image convention. The sudden jumps in the dihedral angle due to these imaging issues then resulted in the decoupled ligand, even though restrained, leaving the binding site. Of course, this was simply an artifact of periodicity—but because of the handling of periodicity in the calculation of restraints in GROMACS, this resulted in sudden jumps in restraint energy/forces and caused problems. Thus, we adjusted our restraints selection procedure to avoid this problem.

Therefore, for the first protein atom, the algorithm takes all of the protein atoms that came out of the filtering process described above and picks the first atom (P1) where the angle P1–L1–L2 between that protein atom and two of the ligand atoms is at least 10 kT from 0 or 180° (see eq 2), where the dihedral angle P1–L1–L2–L3 is between −150 and 150°, and where the circular variance (as implemented in the SciPy package in Python) of that angle and dihedral angle are smaller than 100 or 300 deg^2^, respectively.

For the second protein atom, P2, the algorithm picks an atom that is at least 0.5 nm away from the first protein atom but no more than half the box size and that passes the same angle (P2–P1–L1) and dihedral angle (P2–P1–L1–L2) checks as described above.

For the third atom P3, the algorithm picks the protein atom that is farthest from P1 and P2 but no more than half the box size away from them and where the dihedral angle P3–P2–P1–L1 passes the same checks as above. The same protein atoms are used for restraining both ligands if selected atoms pass the above checks in both systems. If the protein atoms selected for one protein–ligand complex are not suitable for the other protein–ligand complex, the algorithm selects different protein atoms for the second system. Ligand atoms, on the other hand, are selected independently for each ligand.

After the algorithm selects the six atoms for the restraints, the equilibrium position values for the bond, angles, and dihedrals are calculated either from a single input structure or, if a trajectory is provided, from all provided frames, and the mean value is used for the restraints.

We also found that the equilibrium length of the distance restraint has an impact on the mobility of the ligands, meaning that the chosen restraint force constant should vary with the distance restraint length chosen (if a constant level of ligand motion is the goal). In particular, the arc length *s* the ligand can move along around the surface of a sphere, where *s* = *r*θ and *r* is the distance and θ the angle P1–L1–L2, scales roughly quadratically with the distance. Therefore, we increase the force constant of that angle restraint quadratically with the distance.

## 3 Systems

We evaluated the performance of SepTop on four pharmaceutically relevant test systems.

**Figure 4:**
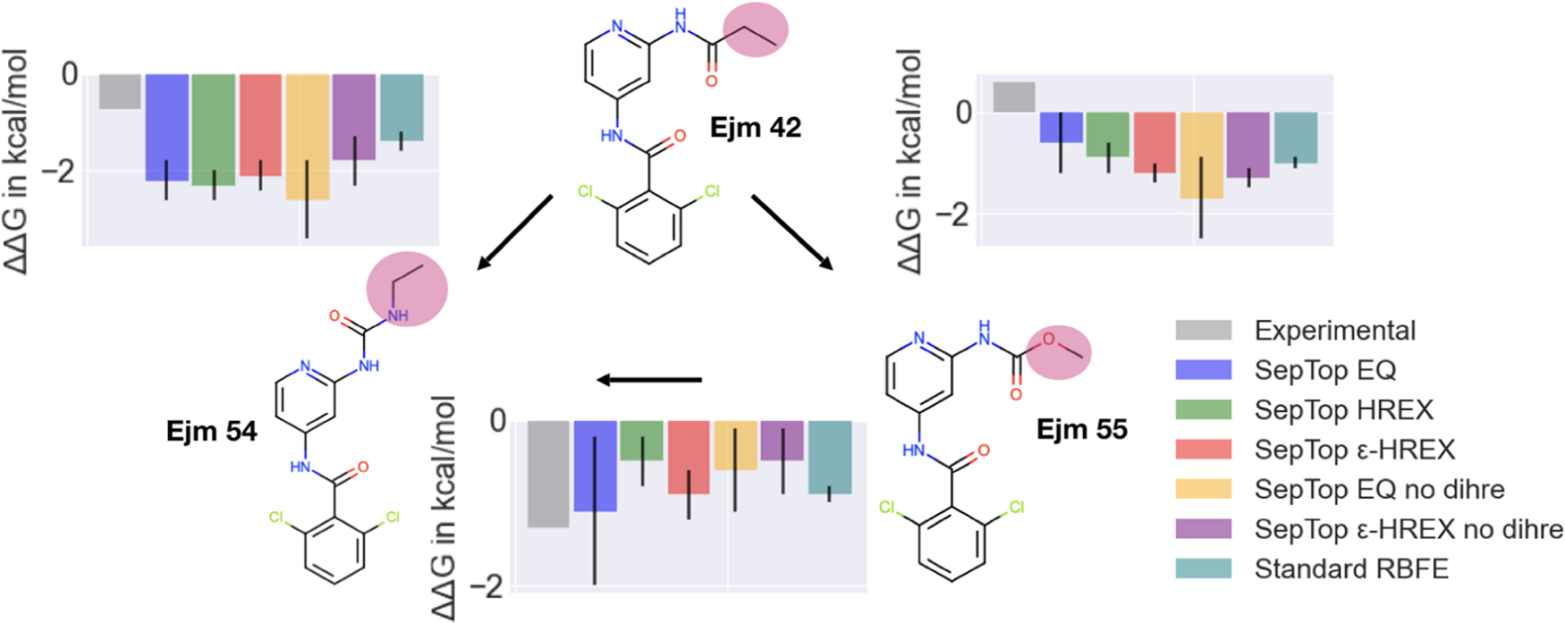
Perturbation cycle in the TYK2 system. For all three transformations, we report the experimental (gray) and calculated relative binding free energies. In the SepTop approach, five different protocols were tested, two without enhanced sampling (EQ and EQ no dihre), one using HREX, and two protocols using ϵ-HREX. In the first three protocols, rotatable bonds were restrained using dihedral restraints, while in the fourth and fifth protocols (purple and yellow), no dihedral restraints were applied. The last protocol (teal) shows values obtained from the work of Ge et al. using standard RBFE.^42^ SepTop and standard RBFE produced similar results. With SepTop, different protocols converged to the same relative free energies within uncertainty, and the standard deviation across trials and the cycle closure error (Figure S1) was lower when using enhanced sampling techniques.

We picked three ligands binding to tyrosine kinase 2 (TYK2) as the first test system.^43^ Those three ligands were structurally very similar and differed by small R-group changes (Figure 4). This allowed us to compare SepTop to standard RBFE, making this system a good sanity check to ensure that the method is giving correct results. Using the same input structures and force field parameters from a previous study,^42^ the ΔΔ*G* values of different RBFE methods should converge to the same result within statistical uncertainty.

**Figure 5:**
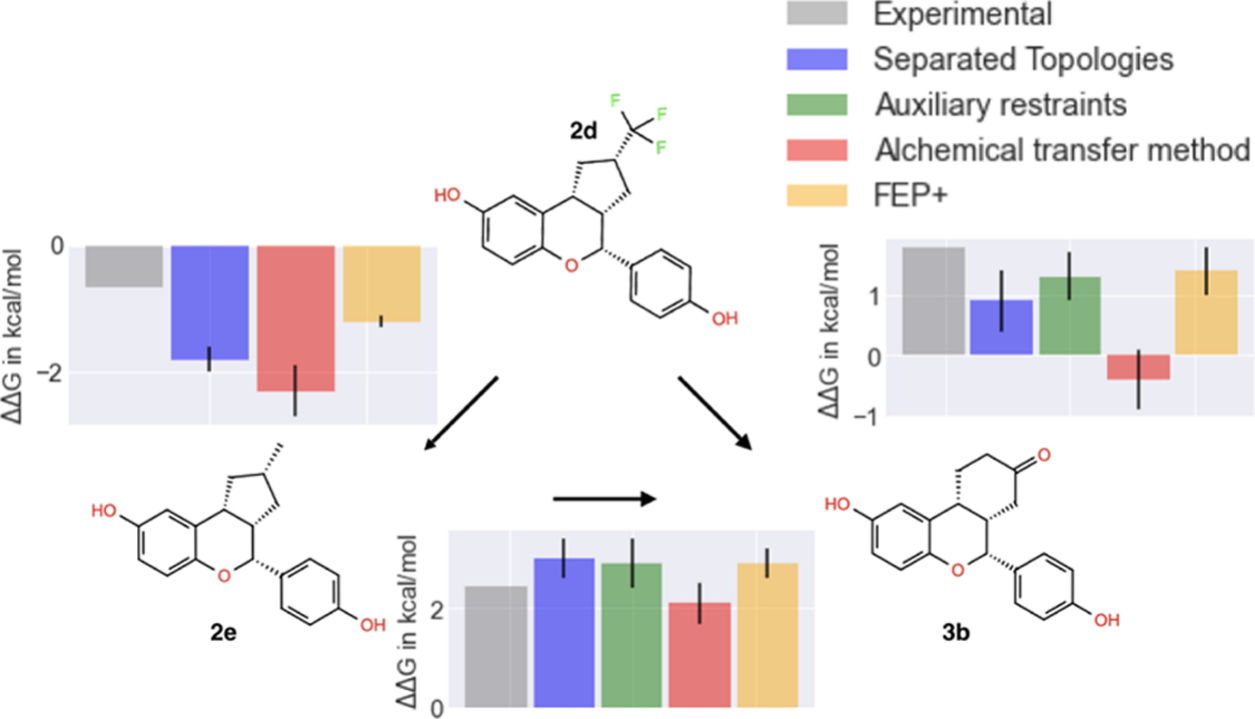
Ligand cycle in the ERα system. For all three transformations, we report the experimental and calculated relative binding free energies. The error estimate in SepTop is the standard deviation calculated across three independent repeats. Overall, different methods gave similar results; however, they did not always converge to the same ΔΔ*G* values.

Estrogen receptor α (ERα) systems have been studied by multiple groups for scaffold hopping transformation and were therefore an interesting next test system for SepTop. The transformations involve ring extensions; here, in particular, the key ring change is a transformation from a five to a six-membered ring (Figure 5). These scaffold hopping transformations fall outside the scope of regular RBFE, although they can be calculated using a soft-bond potential,^19^ additional restraints,^20^ or the alchemical transfer method.^36^ Here, we wanted to investigate the performance of SepTop on challenging transformations like these and compare results with other methods.

We then tested the approach on a larger dataset of 16 ligands binding to mucosa-associated lymphoid tissue lymphoma translocation protein 1 (MALT1). The ligands mostly differ by small R-group changes; however, there are a few ring formation transformations (isopropyl to cyclopropyl) and one stereo inversion transformation (see Figure S1) that can pose challenges in standard RBFE if the chimeric molecule (the molecule that is composed of atoms to represent both ligands) is not created carefully.

**Figure 6:**
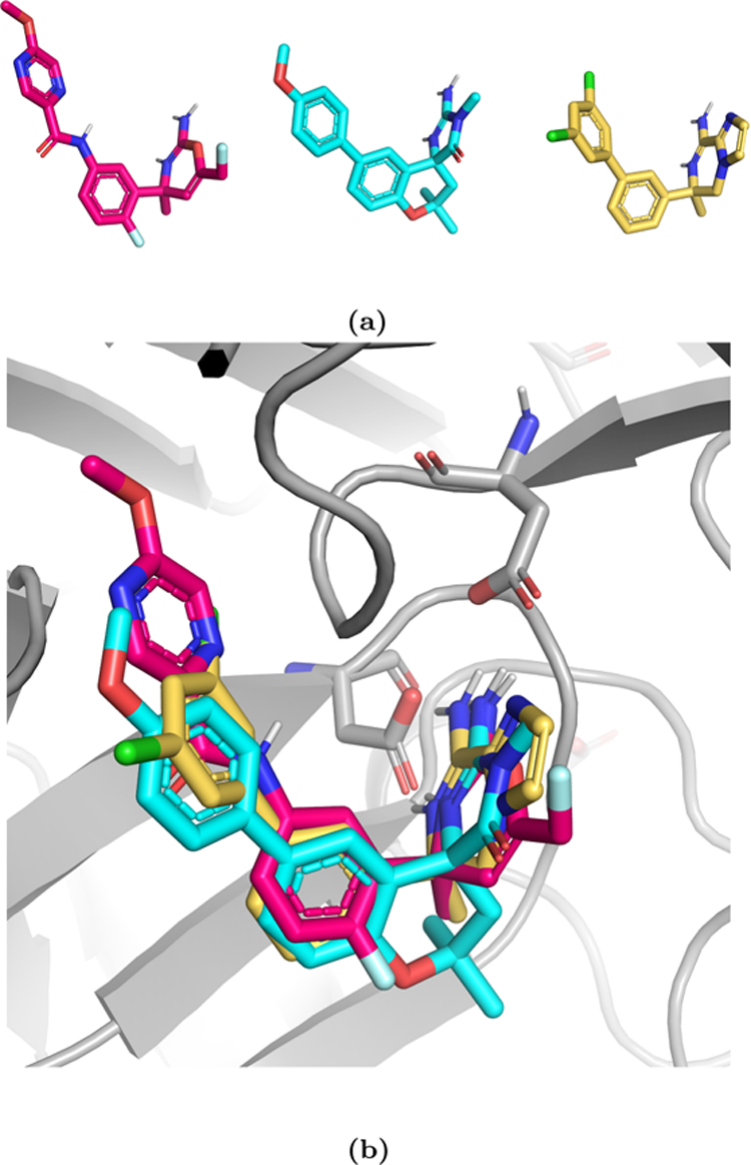
Three scaffold series in the BACE1 system. (a) A ligand from the amide series is shown in magenta, the spirocyclic series in blue, and a ligand from the biaryl series in yellow. (b) Overlay of the three scaffolds in the binding site. The catalytic aspartate dyad is shown in the sticks. The three ligand scaffolds are diverse, e.g., the pyrazine ring of the ligand from the amide series extends into the P3 pocket of BACE1.

Lastly, we tested the performance of SepTop on charged ligands with diverse scaffolds binding to β-secretase 1 (BACE1). BACE1 has been used as a benchmark system for free energy calculations.^44,45^ Different scaffold series of BACE inhibitors have always been treated separately, meaning that separate RBFE maps were run for each scaffold.^6,46^ Here, we tested SepTop on ligand transformations both within different scaffold series and also across different series. This dataset and those scaffold hopping transformations would be very challenging using standard RBFE methods making this a good test case for the domain of applicability of SepTop. We ran calculations for three different ligand series, the amide series, a spirocyclic series, and a biaryl series (Figure 6). Different series conserve the interaction with the catalytic aspartate dyad, while the rest of the scaffolds are diverse. Especially the amide series, having a longer linker, extends into the P3 pocket, displacing some water molecules that are present in the two other series. We chose six ligands per series and performed calculations both within each series as well as across different series.

## 4 Simulation Details

In the following section, we describe the preparation of the proteins and ligands of the systems in this study. We then go over the setup of the systems for SepTop, as well as provide details about running and analyzing the free energy calculations.

### 4.1 Preparation and Parametrization of Proteins and Ligands

The proteins and ligands for the four systems in this work were prepared in order to generate input structures for the MD simulations. Topology and coordinate files of all systems can be found in the Supporting Information (SI).

For the TYK2 system, we selected three ligands from the protein–ligand Benchmark set (ligand codes: ejm 42, ejm 54, ejm 55 as defined in the Benchmark set^45^). We obtained the input coordinates and force field parameters from the protein–ligand benchmark set. Using the same input structures and parameters as in the previous study by Ge et al.^42^ allows for a comparison of SepTop and traditional RBFE, specifically the efficiency of the free energy perturbation independent from the effect of the force field or system preparation. The ligands were parameterized with Open Force Field version 1.0.0 (codenamed “Parsley”)^47^ and AM1-BCC charges.^48^ The AMBER ff99sb*ILDN force field^49^ was used to parameterize the protein, and the TIP3P model^50^ was used for the water. GROMACS^37,51^ was used to solvate the ligands and the protein–ligand systems and to add ions to reach a salt concentration of 150 mM. The output topology and coordinate files were then used to create the input files for SepTop.

For the ERα system, we ran calculations using structures prepared in three different ways. We prepared the first structure ourselves using a protocol we detail below. The other two input structures were used in the studies of Azimi et al.^36^ and Zou et al.^20^ and were provided by the authors. These systems were parameterized using Gaff1.8,^52^ Amber ff14SB,^53^ and TIP3P water.^50^ To prepare our own structure, we began with PDB 2Q70. We performed the structure preparation on OpenEye’s Orion cloud computing platform, using their workflow (”floe”) “SPRUCE-Protein Preparation from PDB Codes” with the default parameters.^54^ In this workflow, hydrogen atoms were added, missing loops were built, and crystallographic waters were retained. The two chains of the homodimer were separated, and chain A was used for the rest of the study. The binding mode and coordinates of the ligands were obtained from the SI of Zou et al.^20^ The prepared protein was aligned onto the protein provided by Zou et al. to be in the correct reference frame for the ligand coordinates. Orion floes “Bound Protein–Ligand MD” for the protein–ligand complex and “Solvate and Run MD” for the unbound ligands were used to solvate, parameterize, and equilibrate the systems. Ions were added to each to achieve a salt concentration of 50 mM. The GAFF1.81 force field^52^ and AM1-BCC charges^48^ were used for the ligands, Amber ff14SB parameters^53^ for the protein, and TIP3P^50^ for the water. In the Orion floes, the systems were energy minimized, equilibrated, and then a production run of 2 ns was performed. The trajectory of that simulation was then used to create the input files for SepTop.

For the MALT1 system, we used PDB 7AK1 as the input structure since the ligand in that crystal structure was similar to the ligands in this study. We prepared the protein using OpenEye Spruce (Orion floe “SPRUCE-Protein Preparation from PDB Codes”). Missing loops were built, and crystallographic waters were retained. The 16 ligands were then aligned onto the crystallographic ligand using OpenEye’s ShapeFit method^55,56^ as implemented in the SystemBuilder package.^57^ Similarly, as described above, Orion floes “Bound Protein–Ligand MD” for the protein–ligand complex and “Solvate and Run MD” for the unbound ligands were used to solvate and parameterize the systems. The Open Force Field version 1.3.1^58^ and AM1-BCC charges^48^ were used for the ligands, Amber ff14SB^53^ for the protein, and TIP3P^50^ for the water.

For the fourth system, BACE1, we chose PDB 6OD6 as the input structure. We prepared the protein as described above using OpenEye Spruce. Chain A of PDB 6OD6 was then used for further simulations. The ligand SDFiles from the amide and the biaryl series were selected from previous Janssen reports and had measured bioactivities from the same assay, while ligand input files from the spirocyclic series were obtained from the protein–ligand Benchmark set with measured bioactivities from a different assay.^59^ The starting binding modes for the different scaffolds were obtained by overlaying the structures onto a crystallographic ligand using the SystemBuilder package as described above. For the amide series, PDB 6OD6 and its crystallographic ligands were used, while for the spirocyclic series, PDB 4JPC was used as input for the ShapeFit algorithm after aligning the PDB structure onto PDB 6OD6. PDB 3IN4, aligned onto PDB 6OD6, was used for the biaryl series. Different PDB structures were used in this step in order to perform the structural alignment onto a reference ligand that was most similar to a particular scaffold series. However, all protein–ligand complexes from different series were then prepared for MD simulations using the same protein structure, PDB 6OD6. In the bound state, the BACE1 catalytic aspartates Asp32 and Asp228 were both treated in their ionized forms. For some ligands, multiple potential binding modes had to be considered. For non-symmetric substituted phenyl rings, we performed SepTop calculations between different orientations of the R-groups, and the more favorable binding mode was then used for further calculations. The solvated ligand and protein–ligand systems were then created with the Orion floes as described above using the Open Force Field version 2.0.0^58^ and AM1-BCC charges^48^ for the ligands, Amber ff14SB^53^ for the protein, and TIP3P^50^ for the water. Sodium and chloride ions were added to reach a salt concentration of 150 mM.

### 4.2 Setup of SepTop Systems

The solvated and parametrized systems were then further prepared for SepTop using a set of Python scripts. Python scripts for performing these operations are currently housed in the GitHub repository SeparatedTopologies,^41^ which is under active development. This package contains multiple Python scripts that can be used to generate all necessary input files for running SepTop in GROMACS, namely

- a coordinate file with the solvated protein–ligand complex having both ligands in the binding site,
- the topology files, including the details for the orientational restraints, and
- the input topology and coordinate files for calculations in the solvent.

The package takes as input the topology and coordinate files of the solvated protein–ligand complexes and the ligands in solution as well as a coordinate file for each of the two ligands (e.g., in the MOL2 or SDF format), and optionally a trajectory of an equilibrium simulation for assisting with the Boresch Restraint setup (see Section 2.2).

#### 4.2.1 Coordinate Preparation and Topology File Generation

This package performs a number of steps to set up each FEP transformation. First, for every transformation (or edge), it aligns the coordinates of the two protein–ligand systems using OpenEye Spruce.^54^ Then, it inserts the coordinates of ligand B into the coordinate file of the protein–ligand A complex, giving a coordinate file with both ligands present in the binding site. As a default, in a transformation from ligand A to ligand B, the protein structure in complex with ligand A is used for the simulations; however, this can be easily adapted by the user to insert ligand A into the protein–ligand B complex.

In the next step, the script creates the topology files needed to perform all transformations in the thermodynamic cycle. Two topology files are generated to describe all transformations in the binding site. This is necessary due to our multi-step alchemical pathway that first perturbs some of the vdW interactions (Figure 1 leg B), then the electrostatics (leg C), and finally, the rest of the vdW (leg D). The first topology file describes leg B and leg C in the thermodynamic cycle, and the second topology file describes the end states in leg D. The respective end states of the transformations were defined as an A and a B state in the [moleculetype] section of the combined ligands in the topology files. We will describe the details for generating the topology files below. Example output topology files are provided in the SI.

To generate the topology files, the script first inserts the topology of ligand B into the topology of the protein–ligand A complex. Then, the [atomtypes] section is modified by adding atom types for dummy atoms that describe the non-interacting ligand, as well as “scaled” atom types, where the LJ-ϵ (see eq 4) was scaled down by multiplying the LJ-ϵ by a scaling factor γ. The later atom types were then used for the enhanced sampling protocol, as described below. In addition, the atom type names for the ligand atoms were renamed to produce distinct atom type names for ligand A and ligand B. This was necessary in order to be able to exclude interactions between the two ligands in the next step by defining special nonbonded interactions between atom types. More specifically, the script adds a [nonbond_params] section to the topology file in which the vdW interactions between atom types of ligand A and atom types of ligand B are defined as zero.

The Coulomb interactions between the two ligands did not have to be excluded since, in this thermodynamic cycle, there are no end states where both ligands have electrostatic interactions turned on at the same time. As implemented in GROMACS, the Hamiltonian of an alchemical intermediate state is constructed by the linear interpolation of the Hamiltonians rather than charges, i.e., *H* = (1 – λ) × *H*_0_ + λ × *H*_1_, where λ is the alchemical parameter, *H* is the Hamiltonian of the alchemical state, *H*_0_ is the Hamiltonian of end state A, and *H*_1_ is the Hamiltonian of end state B. This means that even though both ligands may have partial electrostatic interactions at the same time, the ligands will not interact with one another at any state of the alchemical transition as long as the ligands are not interacting with each other in the end states. The vdW interactions between the two ligands, however, have to be excluded since there are end states in the alchemical path (both end states in leg C, Figure 1) where both ligands have vdW partially or fully turned on.

#### 4.2.2 Setup for ϵ-HREX

We scaled down the LJ parameters, which, combined with Hamiltonian Replica Exchange (HREX),^60−62^ can enhance the sampling of slow degrees of freedom, as has been shown in the implementation REST2^63^ and ACES.^16^ Instead of running different replicas at different temperatures, as in temperature replica exchange, all replicas are run at the same temperature while the potential energy of every replica is scaled differently. This can lead to an increase in the “effective” temperature of the system in the region where the interactions are being scaled, and it was shown that this can improve the sampling efficiency compared to actual temperature replica exchange.^63^ Both nonbonded and bonded parameters can be scaled down to enhance the sampling; however, here, we only scale down the LJ-ϵ parameters of the ligands since we found that this was sufficient to improve sampling in most systems. In some cases, we additionally scaled down the force constant of dihedral angles in the ligand to enhance slow rotamer sampling (see Section 5.4). We will refer to this protocol, as it modifies the LJ-ϵ and performs HREX as ϵ-HREX throughout this work. The LJ potential, *V*_LJ_(*r*), is defined using an ϵ and a σ parameter
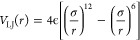
4where *r* is the distance between two atoms.

The modification of the LJ parameters was implemented by adding new “scaled” ligand atom types. We multiplied the ϵ parameter of all original ligand atom types with a scaling factor γ to soften the potential. In this work, we used a scaling factor of γ = 0.5; however, we found that in some cases, it was necessary to further reduce the value to γ = 0.01 in some parts of the molecule in order to achieve sufficient sampling (see Section 5.3).

We adapted the thermodynamic cycle to incorporate this enhanced sampling method. In leg B of the thermodynamic cycle (Figure 1), instead of fully turning on the vdW interactions of the green ligand, vdW interactions were only partially turned on to the modified LJ-ϵ (scaled atom type as described above). At the same time, the vdW interactions of the magenta ligand are partially turned off by transitioning from the original atom type to the scaled atom type. Both ligands have part of their vdW interactions turned on, and therefore, in the new leg D of the ϵ-HREX protocol, the vdW interactions of the magenta ligand are now fully turned off, while the vdW interactions of the green ligand are fully turned on. We compared the performances of this ϵ-HREX protocol with a protocol that did not use enhanced sampling in Section 5.1.

#### 4.2.3 Restraint Atom Selection

In the next step in setting up the topology files, atoms for the Boresch-style restraints were selected according to the algorithm as described in Section 2.2. The restraints are defined in .itp files and added to the corresponding topology files. In this work, a force constant of 20 kcal mol^–1^ Å^–2^ was used for the bonds, and 20 kcal mol^–1^ rad^–2^ for one of the angles and three dihedrals. The force constant for the other angle varied depending on the bond distance. We set it up such that for a bond distance of 5 Å, a force constant of 40 kcal mol^–1^ rad^–2^ would be used for that angle restraint, and with increasing distance, that force constant was scaled quadratically. The contribution for releasing the orientational restraints when the ligands are non-interacting (Figure 1 leg A and leg E) was calculated analytically using eq 32 from Boresch et al.^34^

### 4.3 Setup for Calculations in the Solvent

For calculations in the solvent, we used two different protocols depending on whether the ligands have a net charge. If all ligands in the dataset were neutral, we performed absolute hydration free energy calculations. If ligands were charged, we performed an analogous “separated topology” hydration free energy calculation, where the solvation of the two ligands was performed simultaneously in opposite directions. (As a test of this approach, we transformed a charged ligand into itself in solution and confirmed that the computed free energy converged to zero.) The two approaches required different scripts to generate the respective topology and coordinate files. For the absolute hydration free energy calculations, the tool writes a topology file with a fully non-interacting ligand in the B state. For the charged ligands in the BACE1 dataset, we ran SepTop calculations for the solvent part of the thermodynamic cycle in order to preserve the net charge throughout the simulation. We restrained the ligands such that they remain half the box edge away from each other using a single harmonic distance restraint between the heavy atoms closest to the COM of the two ligands using a force constant of 2.4 kcal mol^–1^ Å^–2^. One ligand was then fully decoupled while at the same time, the other ligand was fully coupled using a similar protocol as in the binding site with the difference that in the solvent, the LJ-ϵ was scaled with a scaling factor γ = 0.3 rather than γ = 0.5 as in the binding site. The system was first equilibrated for 10 ps in the NVT ensemble at a force constant of 0.0024 kcal mol^–1^ Å^–2^ to allow the ligands to gently adjust to the distance restraint, followed by a 10 ps equilibration in the NVT ensemble at the full restraint force constant.

### 4.4 Running SepTop in GROMACS

All MD simulations were performed using GROMACS 2021.2.^37,51^

For simulations in the binding site, we used an alchemical path with a total of 20 λ windows, 8 states for leg B, 5 for leg C, and 8 for leg D. Leg B and leg C were run in one step, meaning that a single topology file and therefore only two end states were used for leg B and leg C together. This combined step then used 12 λ windows, making this a total of 20 λ windows. The details of the alchemical path can be found in the free energy calculations section of the MDP files provided in the SI.

For the unbound part of the thermodynamic cycle, we performed absolute hydration free energy calculations for all neutral ligands. Here, we used 14 λ windows, first turning off the Coulomb interactions and then the vdW interactions of the ligand. For the charged ligands in the BACE1 dataset, we ran SepTop calculations (to avoid changing the formal charge of the system^31^) in the solvent using a total of 28 λ windows (see MDP files in the SI for details); essentially, this involved 14 for each ligand.

All λ windows were first energy minimized using steepest descent for 5000 steps and then equilibrated in the canonical ensemble for 10 ps at 298.15 K. A production run of 10 ns per λ window was performed in the NPT ensemble at a pressure of 1 bar. The MD simulations were performed using the stochastic dynamics integrator at a timestep of 2 fs. The soft-core potential from Beutler et al.^64^ was applied to avoid instabilities in intermediate λ windows. Replica exchange swaps were attempted every 200 steps. Full details of simulation parameters can be found in the MDP files in the SI.

In the TYK2 system, we restrained rotatable bonds in the ligand using dihedral restraints after noticing that slow rotamer sampling led to poor convergence. We adapted the thermodynamic cycle to account for the contribution of these restraints. In step A (Figure 1), the non-interacting ligand B (green) was inserted from the standard state having all its rotatable bonds restrained. Then, dihedral restraints on the interacting ligand A (magenta) were turned on simultaneously with the Boresch-style restraints (step B). The next step is identical to that in the original protocol, except that it has dihedral angles on both ligands restrained. In step D, the restraints on ligand B (green) are released, and in step E, the non-interacting ligand A, with its rotatable bonds restrained, was transferred to the solvent. The interactions of ligand A were then turned back on in the solvent, and the dihedral restraints were released. Similarly, in the hydration free energy calculations of ligand B, first, the dihedral restraints were turned on, followed by the decoupling of the ligand, and then non-interacting ligand B was inserted into the binding site in step A, as described above.

Restraining rotatable bonds can lead to faster convergence in some cases; however, it can also lead to slower convergence of the free energy estimate in other cases. If, e.g., the dihedral angle rotates freely in the solvent, releasing dihedral restraints in the solvent can require the addition of λ windows to obtain sufficient overlap of the work distributions. Therefore, our general SepTop protocol does not restrain rotatable bonds.

### 4.5 Analysis of the Results

The free energy difference was obtained from simulation data using the MBAR estimator^65^ as implemented in the alchemlyb interface to the pymbar package.^65,66^ The first nanosecond of the 10 ns production run was discarded as additional equilibration. In the TYK2, ERα, and BACE1 systems, we ran three independent repeats to assess the convergence of the free energy estimate and to get an estimate of the uncertainty, though not in the MALT1 system to reduce compute costs and to get a better idea of typical production-level accuracy. In the MALT1 and BACE1 systems, absolute binding free energies were obtained from the ΔΔ*G* values using a maximum likelihood estimator as implemented in the arsenic package^67^ (later renamed to cinnabar). That same package was used to generate correlation plots and the error and correlation statistics used in this paper.

## 5 Results

In this study, we investigated the performance of an alternate approach for RBFE, SepTop, on several pharmaceutically relevant systems–specifically, TYK2, ERα, MALT1, and BACE1. Our focus here is on validating the method for such systems rather than the model system studied previously^33^ and on ensuring that the approach is relatively robust and accurate across several different representative targets.

### 5.1 We First Tested SepTop on the Well-Studied TYK2 Dataset as a Sanity Check

The TYK2 ligands that we investigated here only differ by small R-group changes, which allowed us to compare SepTop with standard RBFE to ensure that results are reasonable on a system where we can obtain correct binding free energies for the model with a well-established method. Using the same input structures and force field parameters as in a previous study,^42^ SepTop and standard RBFE using a non-equilibrium switching (NES) protocol gave similar relative binding free energies for three transformations in the TYK2 system. A low cycle closure error of 0.0 ± 0.5 kcal mol^–1^ (SepTop ϵ-HREX Figure 4) suggests that ΔΔ*G* estimates may be well converged, though the low cycle closure does not necessarily imply convergence or an absence of sampling problems since there might be cancellation of errors.

After finding that the SepTop approach worked well on this test system, we used this system as a test case to help us optimize our protocol and increase its efficiency. We found that running each λ window for 10 vs 20 ns gave relative free energies, which were statistically indistinguishable, so we decided to use a simulation time of 10 ns per λ window in future systems. Our initial protocol used 45 λ windows for calculations in the binding site, but we found that we could reduce the number of windows to 20 while retaining good overlap between neighboring states. We used the overlap matrix as implemented in alchemlyb(66) to help find a reasonable alchemical pathway and spacing with sufficient overlap.

We also investigated whether the use of enhanced sampling improved the convergence and efficiency of the simulations. We compared three protocols, one without enhanced sampling, another using HREX, and a third protocol, ϵ-HREX, where we scale down the LJ-ϵ by a factor of γ = 0.5. Scaling down the LJ-ϵ can soften interactions and lower energy barriers between conformational states and can therefore be coupled with replica exchange swaps, improving sampling. All three protocols converged to statistically the same relative binding free energies (Figure 4). The standard deviation, calculated across three independent repeats, was highest when no enhanced sampling was used, while the SepTop HREX and SepTop ϵ-HREX protocols had similar values for the standard deviation. The cycle closure (summation of ΔΔ*G* values along the cycle) was lowest for the protocols that used ϵ-HREX (0 kcal mol^–1^) (Figure S1, suggesting that those calculations may have converged best). Here, we were primarily focusing on comparing results from different methods with one another rather than comparing experimental binding affinities because methodological improvements do not always improve agreement with the experiment. In particular, agreement with the experiment is not just a function of sampling but of multiple factors, such as the force field and the choice of protonation states, counterions, *etc.*, meaning that the best method may not necessarily agree best with the experiment except when all other issues have been addressed.

Restraining all rotatable bonds in the ligands increased the efficiency of these simulations, suggesting that sampling of different rotamers might be slow in this system. The standard deviation of the protocols that used dihedral restraints was overall lower than in the two protocols where the rotatable bond was not restrained (Figure 4). The thermodynamic cycle was adapted accordingly to account for the contribution of the restraints (Section 4). When restraining rotatable bonds in free energy calculations, calculations are likely most efficient if the restraints are applied to the preferred conformation, if that is known a priori, e.g., from an equilibration MD simulation. If a ligand is restrained to an unfavorable dihedral angle, this would theoretically still be accounted for when releasing the restraints; however, such a choice may lead to slower convergence.

Using an enhanced sampling protocol that softens the vdW parameters and performs HREX (Figure 4SepTop ϵ-HREX no dihre) improved the sampling of different rotamers over the standard protocol (SepTop EQ no dihre), shown by the smaller standard deviation across three independent repeats.

### 5.2 Scaffold Hopping Transformations with SepTop on ERα Systems

ERα has been studied by multiple groups benchmarking different scaffold hopping approaches and therefore appeared to be a good test system for our method, giving us the possibility to compare SepTop with other methods. Here, we compared SepTop with three different RBFE methods, the alchemical transfer method (ATM),^36^ as well as an RBFE method that uses auxiliary restraints for scaffold hopping transformations,^20^ and lastly, FEP+.^19^ For these three ligand transformations, different methods converged to similar relative binding free energies (Figure 5). Using the same input structures provided as in the auxiliary restraints paper,^20^ SepTop converged to the same ΔΔ*G* value within uncertainty as the method using auxiliary restraints in the two edges where calculated values had been reported.

**Figure 7:**
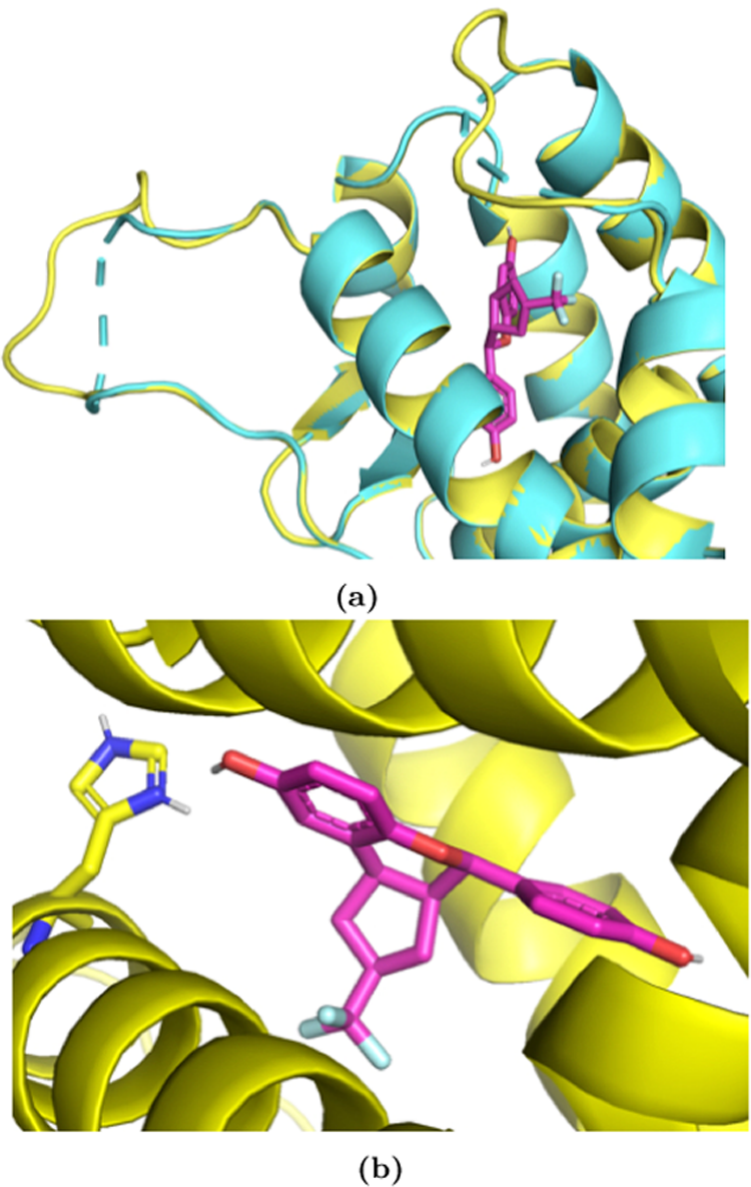
Different choices made in ER α preparation. (a) Two loops were not resolved in the crystal structure, and missing residues were modeled in some studies,^20^ while in other studies, the residues bordering the missing loop were capped and restraints used to keep the protein from drifting apart.^36^ (b) Different software for the assignment of protein side chain protonation states resulted in differences in the protonation state of some histidine residues. One of those residues is located in the binding, and the selected protonation state impacted the ΔΔ*G* estimate, at least in our study.

The comparison of results from different methods was challenging due to differences in system preparation in the different studies. We found that the modeling of missing loops (Figure 7a) and the protonation state of histidine residues (Figure 7b) had an impact on the predicted binding free energy. We ran calculations with the SepTop approach using an input structure we prepared using OpenEye’s Spruce^54^ (Table S1input spruce) and with prepared structures used in the studies of Azimi et al.^36^ (input ATM) and Zou et al.^20^ (input Aux) and obtained very different results (Table S1). When using the input structure that had missing loops, we had to restrain the position of backbone protein atoms to prevent the protein from drifting apart. This could have potentially impacted the results and partly explain why calculations starting from different input structures did not give equivalent results. For one of the transformations (edge 2d-2e), we performed additional calculations to study the impact of the protonation state of histidine on the results. In one protocol, the histidine in the binding site was charged (HIS220, Figure 7b), and in another protocol, HIS220 was neutral, while in one protocol, the protonation state was not reported. We found that the protonation state of that histidine in the binding site affected the calculated ΔΔ*G* by ≈1 kcal mol^–1^. Our finding also shows that depositing prepared protein structures along with a publication is critical for the reprehensibility of results.

### 5.3 We Tested SepTop on a Larger Dataset, 16 Ligands Binding to MALT1

**Figure 8:**
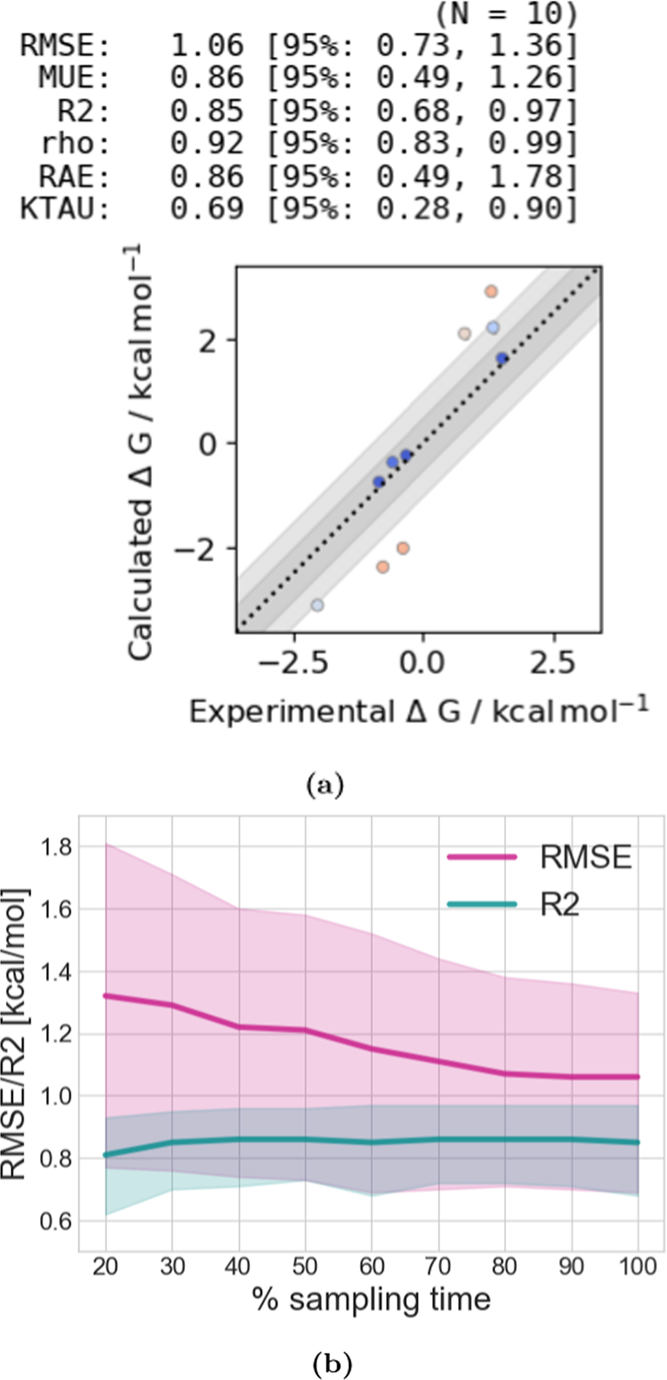
(a) Correlation between predicted and experimental binding free energies for 10 MALT1 inhibitors. The plot was generated using arsenic.^67^ Binding free energies centralized around zero are shown. Root mean square error (RMSE), mean unsigned error (MUE), coefficient of determination (*R*^2^), Pearson correlation coefficient (ρ), relative absolute error (RAE), and Kendall tau (KTAU) are reported with a 95% confidence interval. A good correlation with experimental binding free energies was obtained in this system. (b) Analysis of the RMSE and the *R*^2^ at different amounts of sampling time. The 95% confidence interval is shown as a shaded area. The RMSE remained at a value below 1.1 kcal mol^–1^ after 80% sampling time.

We also tested SepTop on a larger dataset, examining a series of 16 ligands binding to MALT1, and obtained good correlation and error statistics. We created a ligand map in which the 16 ligands were connected with 30 edges. To setup the ligand map, we chose one of the most potent ligands as the reference ligand and connected all other ligands to that reference ligand through edges, creating a star map. We then added additional edges between ligands to create ligand cycles. Absolute binding free energies were obtained from calculated ΔΔ*G* values and experimental binding free energies using the arsenic code.^67^ Since the experimental binding affinities for 6 of the compounds were outside the assay limit, we excluded those ligands from the correlation and error statistics. Correlating the calculated binding free energy of the 10 remaining ligands with the experimental values resulted in a root mean square error (RMSE) = 1.06 and *R*^2^ = 0.85. Additional statistics can be found in Figure 8a. The full set of calculated and experimental values can be found in the SI.

#### 5.3.1 Assessing and Addressing Sampling Problems in the MALT1 System

A cumulative and cycle closure analysis suggested that a sampling time of 10 ns per λ window was adequate for this system. Simulations were mostly converged after 10 ns based on both the confidence interval of the RMSE getting within 0.7 kcal mol^–1^ and the overall low cycle closure errors. We analyzed the correlation and error statistics and the cycle closure at different amounts of sampling time to assess the convergence of the free energy estimate. As seen in Figure 8b, the RMSE decreased with increasing simulation time until reaching a value of RMSE ≈ 1.1 kcal mol^–1^ after around 80% sampling time, while the *R*^2^ remained at a value of around 0.85 after 30% sampling time. Since the RMSE stopped decreasing after 80% sampling time, we concluded that simulations had likely converged.

Assessing how well ΔΔ*G* values in a closed ligand cycle sum up to zero (cycle closure) is an additional metric for convergence. If the edges in a closed ligand cycle do not sum up to zero, simulations are not converged. However, if a cycle closes to zero, this does not necessarily mean that there are no sampling problems since there might be a cancellation of errors. Here, we normalized the cycle closure to obtain an approximate contribution of a single edge to the cycle closure error
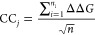
5where CC_*j*_ is the cycle closure of cycle *j* and *n* is the number of edges in cycle *j*. For the cycle closure analysis, we considered the entire ligand dataset, including the compounds with measured binding affinities outside the assay limit. Ligand cycles were enumerated using functions from the NetworkX package,^68^ which were modified by prior authors^69^ to handle this problem. Most of the ligand cycles (51/87) had a cycle closure below 0.5 kcal mol^–1^; however, for 6 ligand cycles, the cycle closure was above 1 kcal mol^–1^, indicating sampling problems (see Figure S3).

We introduced a voting system to help identify bad edges, meaning transformations that likely have not converged yet. Even this relatively small dataset of 16 ligands resulted in 87 different ligand cycles, which made it difficult to identify which specific transitions were unconverged by manually looking at the cycle closure errors. Our voting system provided a more automated way to assess this. In this voting system, if a cycle had a cycle closure above a certain threshold (0.7 kcal mol^–1^), each transformation in the cycle was assigned a penalty (+1), while each transformation in a cycle with a cycle closure below a certain threshold (0.3 kcal mol^–1^) received a positive vote (−1). The votes for each transformation were then summed up, and the edges with the most positive overall votes were investigated further to identify potential sampling problems.

**Figure 9:**
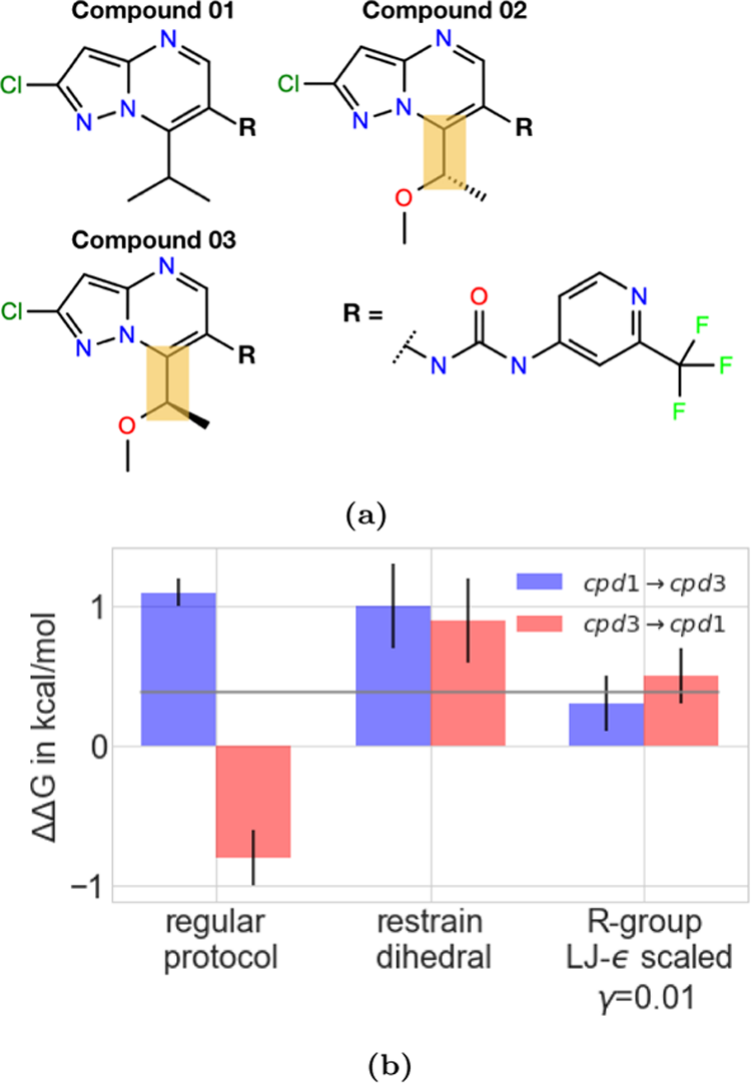
(a) 2D structure of MALT1 ligands, compounds 01–03. The yellow box highlights the rotatable bond whose rotamers were not sampled sufficiently and therefore caused sampling problems (b) ΔΔ*G* values for transformations from compound 01 → compound 03 (blue) and compound 03 → compound 01 (red, here the negative of the ΔΔ*G* is shown) using three different protocols. In the “regular protocol”, the LJ-ϵ of the entire molecules was scaled down by multiplying it with a scaling factor of γ = 0.5. In the “restrain dihedral” protocol, the rotatable bond shown in yellow (a) was restrained during the free energy calculation, and the contribution of the restraint was accounted for by releasing the restraints afterward. In protocol “R-group ϵ = 0.01”, the LJ-ϵ of the ethyl methyl ether group was scaled down by a scaling factor of γ = 0.01, allowing Hamiltonian lambda exchange to help accelerate sampling of this bond rotation. The gray line shows the experimental binding free energy. Using the regular protocol, transformations from both directions do not converge to the same relative free energy due to sampling problems, while transformations in both directions using the two other protocols converged to the same free energies as well as did transformations within the same protocol.

This voting system identified the transformation from compound 03 to compound 01 (Figure 9a) as the worst edge, and indeed, follow-up work showed that this transformation suffered from significant sampling problems. For this transformation, the standard deviation across three independent repeats was low (0.2 kcal mol^–1^). However, running the transformation in the opposite direction (compound 01 → compound 03) gave very different results (ΔΔ*G* = 1.1 ± 0.1 kcal mol^–1^) than the original direction (ΔΔ*G* = 0.8 ± 0.2 kcal mol^–1^, before accounting for the sign flip). This difference of 1.9 kcal mol^–1^, depending on the direction, indicated sampling problems in this edge.

We found that insufficient sampling of different rotamers around the bond highlighted in yellow (Figure 9a) caused these sampling problems. Rotation around a rotatable bond in compound 03 (Figure 9a) was slow in some λ windows and depended on whether this ligand started as a dummy or fully interacting. Analyzing the free energy change caused by modifying the vdW/restraints vs the Coulomb interactions separately showed that the difference in the ΔΔ*G* depending on the direction mostly happened in the λ windows modifying the Coulomb interactions. As described in Section 4.2, replica exchange swaps were carried out between all λ windows in legs B and C in the thermodynamic cycle (Figure 1), while leg D was run in a separate calculation. In the transformation going from compound 03 to compound 01, compound 03 started as a fully interacting ligand, and only a single rotamer of the R-group (Figure 9a) was sampled in all λ windows of leg B and leg C. Meanwhile, when going in the opposite direction, from compound 01 to compound 03, compound 03 started as a dummy ligand in leg B, sampling multiple rotamers. Here, in contrast to the transformation in the other direction, two different rotamers were sampled in the λ windows in which the Coulomb interactions were modified (leg C), benefiting from broad rotamer sampling in non-interacting and weakly interacting states through replica exchange. In this example, the rotamer distribution differed across different states in the alchemical pathway, and since, for some states, the equilibrium rotamer distribution was not sampled, the results were incorrect.

We were able to improve the sampling of different rotamers by scaling the LJ-ϵ down by multiplying it by a scaling factor of γ = 0.01. Free energies for transitions in the two opposite directions now converged to the same result (Figure 9b). Only the LJ-ϵ of atoms in the ethyl methyl ether group (Figure 9a) was scaled down by a factor of γ = 0.01, while for the rest of the molecule, a scaling factor of γ = 0.5 was used. We had tried to scale down the LJ-ϵ of the entire molecule; however, we found that this led to instabilities and convergence problems. We also found that convergence could be apparently achieved by restraining the dihedral of that rotatable bond during the simulation and releasing the restraints afterward. This, however, then led to higher standard deviations and slow convergence when releasing the dihedral restraint in the solvent. In the solvent, different rotamers were sampled in the interacting state, which led to a poor overlap of λ windows when releasing the dihedral restraint (results not shown). Overall, in this case, the protocol which scaled back interactions of the mutated R-group performed best.

#### 5.3.2 Comparing SepTop with a Standard RBFE Method

We compared SepTop with standard RBFE using the non-equilibrium switching (NES) method as implemented in Orion^70^ (Orion floe “Equilibration and Non-Equilibrium Switching”) using the default 6 ns equilibration of the end states and 80 non-equilibrium switching transitions with 50 ps switching time. We used the same ligand map and force field parameters as with the SepTop protocol. We only included ligands with measured binding affinities within the assay limit in our analysis, as we did above.

**Figure 10:**
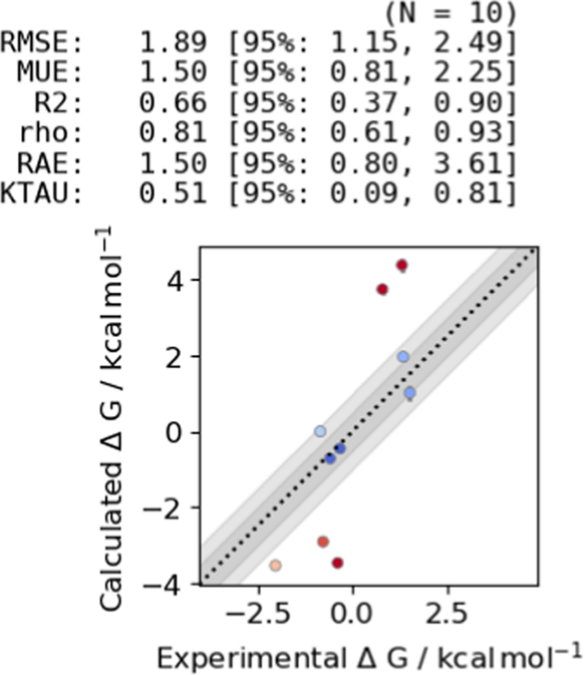
Correlation between calculated (NES Orion) and experimental binding free energies for 10 MALT1 inhibitors. The plot was generated using arsenic.^67^ The correlation and error statistics are worse than the ones obtained using SepTop (Figure 8a). A comparison between NES and SepTop values is shown in Figure S4.

We found that SepTop produced better correlation and error statistics than the more standard NES approach (Figure 10). SepTop used more sampling time than NES Orion (for 30 transformations: bound state: 6000 vs 336 ns; unbound state: 2240 vs 336 ns), which, in addition to the problems detailed below, might be part of the reason why SepTop performed better on this system. Since it would have been cost-prohibitive to increase the length of the simulations on Orion to the simulation time used in the SepTop protocol, we decided not to perform a direct comparison at equal sampling time. The ΔΔ*G* values calculated using the two different methods mostly agree well with one another, as can be seen in Figures S4 and S5. The outliers in the plot indicate that for some of the transformations, the two methods did not agree with each other. Two of those outliers involved transformations going from a hydrogen (Pfizer-01-05) or a methyl group (Pfizer-01-04) to a cyclopropyl (Pfizer-01-07). The overlap of forward and reverse work distributions of the non-equilibrium switching transitions was poor (Figure S6), indicating insufficient sampling of important motions. A third outlier was a transformation that involved the inversion of a chiral center, which was potentially not treated correctly in the NES protocol, as discussed below. For the two outlier transformations Pfizer-01 → compound 02 and Pfizer-01 → compound 03, NES predicted a more dramatic change in potency than SepTop. These ligands contained the R-group that had caused sampling problems due to slow rotamer sampling in SepTop (see Section 5.3.1), which potentially also caused problems in the NES approach.

With the NES approach, some of the transformations in this set can potentially be challenging for standard RBFE calculations if the chimeric molecule is not set up carefully. Those transformations involve stereocenter inversions (compound 02 → compound 03) and ring forming/ring breaking transformations (isopropyl to cyclopropyl transformations). For the latter, chimeric molecules should be created where the entire group is included in the transformation rather than forming/breaking a ring. The chiral inversion transformation was potentially not handled correctly in the NES protocol, possibly due to a bug in the OpenEye implementation.

### 5.4 Testing SepTop on Large Scaffold Hopping Transformations in the BACE1 System

We ran RBFE calculations for three different series of BACE1 inhibitors, each based on a different scaffold, to test the performance of SepTop on large and challenging transformations. We picked six ligands per series and ran calculations within each series as well as spanning between the different series.

#### 5.4.1 In Order to Preserve the Net Charge, We Ran SepTop in Solvent for These Charged Ligands

Since all BACE1 inhibitors in this study were positively charged, instead of running absolute hydration free energy calculations in the solvent leg of the thermodynamic cycle, we performed relative hydration free energy calculations using a similar SepTop approach as in the binding site. Running absolute hydration free energy calculations of charged ligands in the solvent would have led to a change in the net charge of the system (which is difficult to treat for technical reasons^31^), while the net charge can be preserved using a relative approach. The ligands were restrained such that they remain half the box edge away from each other using a single harmonic distance restraint (see Section 4.2). We had first attempted restraining ligands such that they are placed on top of each other using a single harmonic distance restraint; however, we found that this led to slow convergence of the free energy estimate in some cases. We therefore decided to restrain ligands such that they remain far apart.

#### 5.4.2 Running SepTop between Different Binding Poses Can Help Determine the More Favorable Binding Mode

When multiple binding poses were plausible (as determined by overlaying the ligand onto co-crystallized ligands of different PDB structures using a maximum common substructure overlay as well as docking compounds into the site), we ran SepTop between different binding modes to identify the more favorable pose.

**Figure 11:**
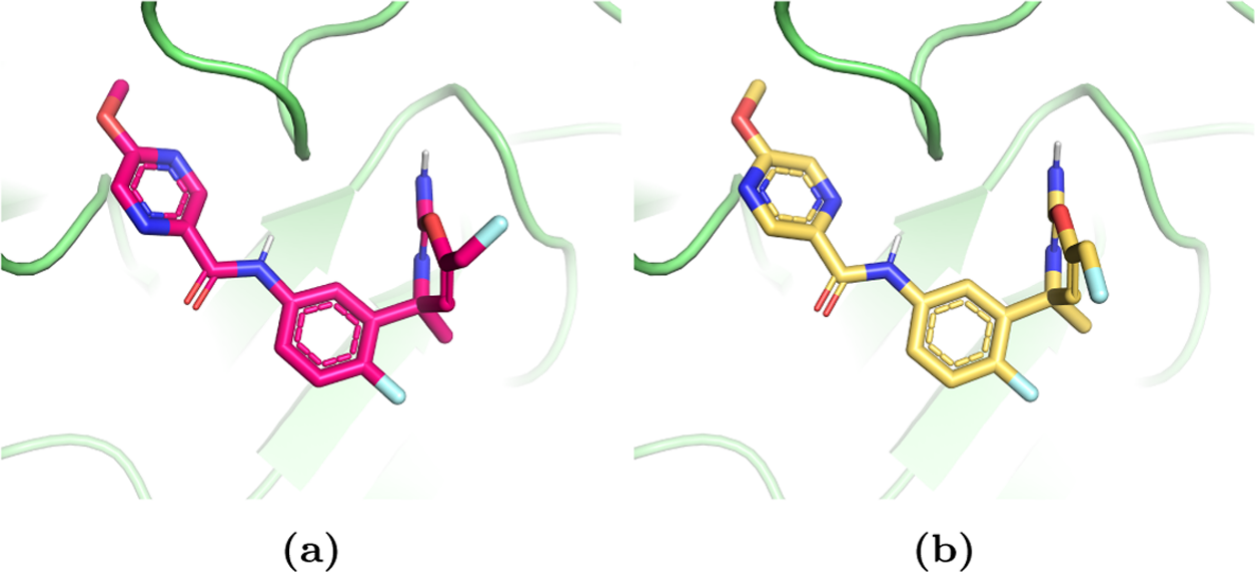
Two poses of a ligand from the amide series binding to BACE1. The orientation of the pyrazine ring in the binding site was unknown. Running SepTop between the two poses showed that the orientation of the pyrazine in the yellow pose (b) was more favorable than the magenta pose (a). The more favorable pose (b) can form an intramolecular hydrogen bond between one of the pyrazine nitrogen and the hydrogen of the amide.

Especially for asymmetric phenyl substituents, the orientation of the ring in the binding site was unknown, and sampling different rotamers was slow such that transitions did not occur during the length of the simulation. In the amide series, different rotamers of the pyrazine ring (see Figure 11) affected computed relative free energies in the binding site by more than 6 kcal mol^–1^. Running SepTop between the two poses differing in the orientation of the nitrogen atoms in the pyrazine ring, as depicted in Figure 11, resulted in a ΔΔ*G*_site_ = −6.6 ± 0.2 kcal mol^–1^ between the two poses in the binding site. This showed that one of the poses (Figure 11b) was predicted to be more favorable than the other pose, possibly due to the potential to form an intramolecular hydrogen bond between one of the nitrogen atoms in the pyrazine ring and the hydrogen on the amide nitrogen, or because the alternate pose places the lone pair of the nitrogen too close to the lone pair of the amide oxygen, resulting in strong repulsion. Transitions between the two poses were only observed in a few λ windows and were very slow. The binding mode with the best docking score was not always the same pose that was predicted to be more favorable in SepTop calculations. For ligands in the biaryl series with an unknown orientation of the asymmetrically substituted phenyl ring, we also ran SepTop calculations between different poses, keeping the binding mode that was predicted to be more favorable for further calculations. In the spirocyclic series, we chose the same orientation of the phenyl rings as given in the binding modes of the ligands in the PLBenchmark set.^45^ The coordinate files of the ligands in their binding modes that were predicted to be more favorable can be found in the SI.

We adapted our alchemical protocol to try to enhance the sampling of different binding modes; however, we found that it was challenging to converge rotamer distributions at all λ windows. Here, our idea was to see whether, instead of running SepTop between different poses to identify the most favorable starting pose, we can start calculations with an unfavorable starting pose and adapt the alchemical protocol to sample the transition to the favorable binding mode. However, in the amide series, the torsion barrier around the rotatable bond between the pyrazine ring and the amide was so high that even in the fully non-interacting state, no transitions between different rotamers were observed. We adapted our enhanced sampling protocol and set the force constants to zero for all torsions that pass through the bond between the pyrazine ring and the carbonyl carbon of the amide in the non-interacting state, as well as further softening LJ-interactions of the atoms forming those torsions by scaling the LJ-ϵ by a factor of γ = 0.1. Starting SepTop from the two different poses using this adapted protocol gave a ΔΔ*G*_site_ = −0.6 ± 0.3 kcal mol^–1^, which was much closer to zero, which we would expect at sufficient sampling. The pyrazine ring now sampled different rotamers; however, sampling was not sufficient in all states along the alchemical path resulting in slow convergence. Thus, while we were eventually able to get this protocol to work, it involved considerable difficulty and manual tuning and still exhibited signs of clear sampling problems, indicating that it would likely not work robustly for other similar problems. Since we sought a general solution, we thus diverted our attention back toward protocols in which the preferred binding mode was an input (even if determined by an earlier SepTop calculation).

#### 5.4.3 With SepTop, We Obtained Good Results for This BACE1 Dataset

To run the SepTop calculations for this BACE1 dataset, we created ligand maps performing transformations both within each series as well as transformations spanning across different series. We ran 10 transformations for the six ligands within each series and 5 transformations between each series pair, giving a total of 45 transformations. Some transformations within a series were also scaffold hopping transformations, such as ring extensions, and we manually created the perturbation map to include both challenging transformations and transformations involving smaller R-group changes.

**Figure 12:**
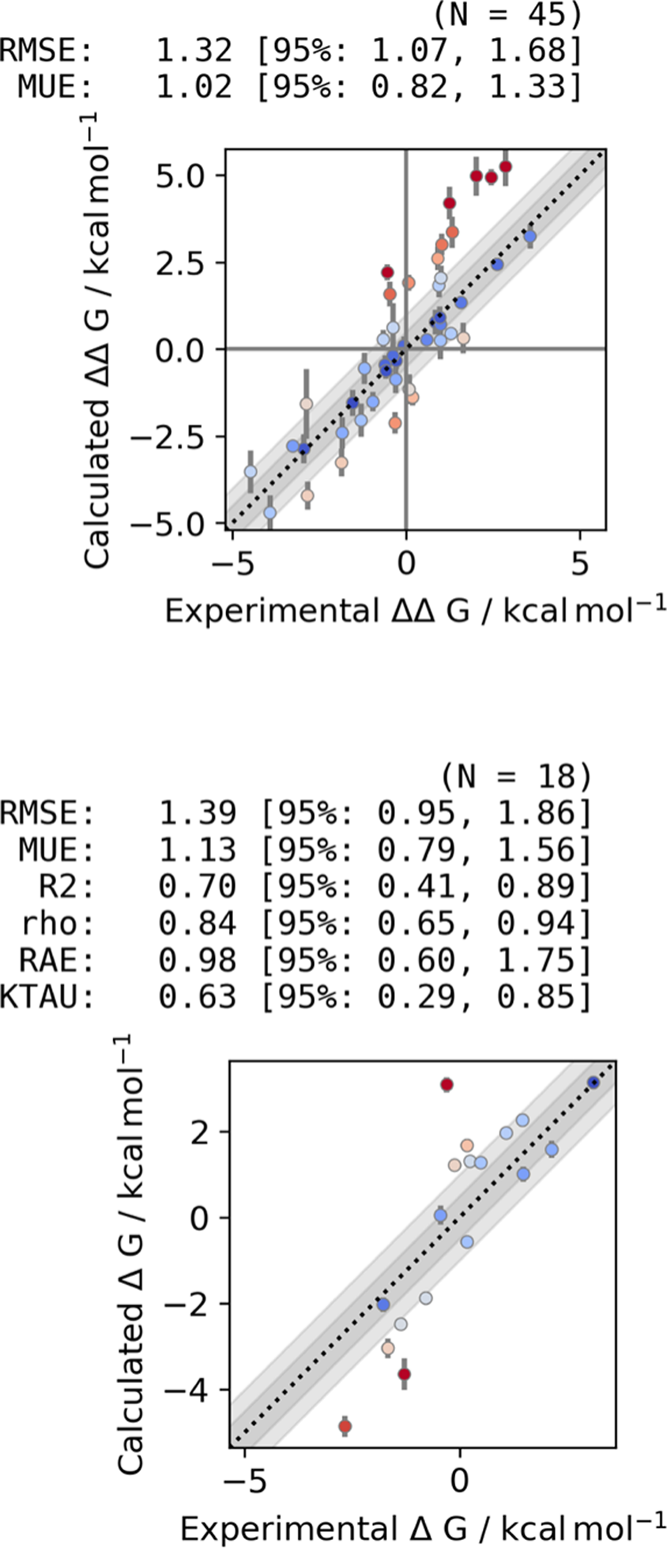
Correlation between calculated and experimental binding free energies for 18 BACE1 inhibitors. The upper plot shows the ΔΔ*G* values of 45 ligand transformations, and the lower plot shows the final calculated absolute binding free energies of the 16 ligands. The plot was generated using arsenic.^67^ Calculated and experimental binding free energies correlated well, given an RMSE of 1.39 kcal mol^–1^ and *R*^2^ = 0.70.

For this dataset, good correlation and error statistics were obtained within different scaffold series as well as for transformations across different series. Figure 12 shows the correlation between experimental and calculated binding free energies of the overall dataset. We show a correlation both for the raw ΔΔ*G* values of the 45 ligand transformations and the Δ*G* values of the 18 ligands. Overall, the correlation with experimental data was good, with an RMSE of 1.39 kcal mol^–1^ and an *R*^2^ = 0.70. There were some outliers with larger deviations from the experiment both within some of the series as well as for transformations between different series, as can be seen in the correlation of calculated and experimental ΔΔ*G* values, broken up by transformations between and within different series in Figure S7. We found that for this system, the ΔΔ*G* values from transformations between ligands within the same scaffold correlated better with the experiment than transformations between ligands from different scaffolds (RMSE = 1.02 vs RMSE = 1.78), which is unsurprising since transformations across scaffolds are clearly more challenging. However, transformations across scaffolds are impossible for standard RBFE calculations, potentially making these transformations still appealing.

We ran three independent repeats of the simulations and reported the mean and standard deviation across those. We then repeated the analysis only considering a single repeat to evaluate the impact of running simulations in triplicates on the results. The correlation between calculated and experimental Δ*G* values was very similar when considering only a single repeat (RMSE = 1.34 and *R*^2^ = 0.70) vs three replicates (RMSE = 1.39 and *R*^2^ = 0.70). The full set of calculated and experimental values can be found in the SI.

We investigated the outliers in this system to try and identify potential sampling problems. All transformations between the amide and the biaryl series had calculated ΔΔ*G* values that were too unfavorable (Figure S8a). Extending the length of the simulation from 10 ns per λ window to 20 ns improved the results slightly (RMSE_ΔΔ*G*_ = 2.23 vs 1.83 kcal mol^–1^), suggesting that simulations had not reached convergence yet. Preliminary free energy calculations initiated from different conformations of the 10s loop (PDB ID: 4FS4 vs 6OD6) showed that results substantially depend on the starting structure since the conformational change was not sampled adequately.

## 6 Discussion and Conclusions

In this paper, we introduce a generalized and efficient Separated Topologies approach and show that it works on pharmaceutically relevant systems. We developed a stable implementation of the approach and provided a package to set up input files for running SepTop in Gromacs.^41^

### 6.1 SepTop Performed Well on the Test Systems in This Study

We tested the method on several diverse, pharmaceutically relevant systems and reported performance and any resulting insights into strengths, weaknesses, and challenges. After validating the method on smaller datasets (TYK2 and ERα), we tested SepTop on two larger datasets, MALT1 and BACE1, with transformations not as straightforward to run with typical RBFE. For both targets, results correlated well with the experiment, and good error statistics were achieved. We analyzed cycle closures as a convergence check and identified sampling problems due to slow rotamer sampling of different dihedral angles in the ligand.

Overall, SepTop performed well for large scaffold transformations in the BACE dataset. These transformations were challenging or impossible for traditional relative free energy calculations for multiple reasons. First, the maximum common substructure shared by the different ligand scaffolds was very small. In addition, in transformations between the amide series and the two biaryl series, compounds from the amide series extended into the P3 pocket of BACE1 and displaced water molecules that were present in the two biaryl series. These large modifications were captured well in this dataset using SepTop.

For transformations between ligands in the same scaffold series in the BACE system, SepTop achieved an RMSE of 1.02 kcal mol^–1^. Two of the three series (the amide series and the spirocyclic series) have been studied using standard RBFE approaches.^7,44,46^ Those studies treated the different ligand series separately and reported an RMSE of around 1 kcal mol^–1^ on each. Thus, on this BACE system, SepTop achieved performance comparable to prior studies; however, this is the first study that carried out transformations spanning across these distinct chemical series, possible here because our approach is more general.

### 6.2 Ways to Test Whether a Target Is within the Domain of Applicability of the BFE Approach

In general, free energy calculations require a particular target to be within the domain of applicability of a method,^5,71,72^ but it is often difficult to know a priori what that domain of applicability is. Thus, when starting to work on a new system, it can make sense to first test the convergence of the free energy estimate on a smaller subset to assess whether a new target is within the domain of applicability of the method. By domain of applicability, we here mean that the binding mode is known and that there are no slow binding mode changes as ligands are swapped, e.g., slow water rearrangements or slow protein conformational changes since the best results will be achieved in general by knowing the preferred conformation/binding mode of the ligand in advance. The convergence of the free energy estimate can be tested with different methods. For example, running calculations in replicates and looking at the standard deviation across repeats, as well as running transformations in opposite directions (*A* → *B* and *B* → *A*), can help assess convergence. Additionally, a high cycle closure in a cycle of ligand transformations is another indicator that an important degree of freedom was not sampled sufficiently. In some cases, more ambitious convergence tests may be beneficial, such as starting simulations from different available protein structures (e.g., structures that had been crystallized with different ligands bound) as well as starting simulations with different water rearrangements in the binding site. If the starting structures impact the calculated binding free energy, convergence has not been achieved yet.

If the binding mode of a ligand is unknown, we have found it to be helpful to run SepTop between different poses to determine the more favorable binding pose, which we then used for further simulations (Section 5.4). We also found that adapting the alchemical protocol by modifying bonded and nonbonded interactions of the ligand (e.g., scaling down the LJ-ϵ (ϵ-HREX) and dihedral force constants) may not be sufficient to sample the correct binding mode sufficiently when starting simulations from the wrong pose (Section 5.4). Based on our results so far, we strongly advise against trying to engage in ligand binding pose prediction while doing free energy calculations, but instead selecting one binding pose prior to the calculation (e.g., by running SepTop between binding poses to determine the more favorable pose).

In some cases, it may be possible to reduce the reliance on starting near the correct binding mode, such as by scaling down the LJ-ϵ of an R-group to enhance rotamer sampling. However, this method needs to be studied on more systems to see whether

1. there is a way to automatically assess which R-group needs to be included in the softening/enhanced sampling protocol and
2. the scaling factor that we picked for the MALT1 system (γ = 0.01, Section 5.3.1) is a good scaling factor for a general protocol.

### 6.3 Comparing SepTop to Other Alchemical Binding Free Energy Methods May Not Be Advisable for Some Systems

If ligands in a dataset are structurally very diverse and fall outside the scope of standard RBFE approaches, in addition to RBFE methods like SepTop, binding free energies can be calculated using standard absolute binding free energy calculations. For some targets, however, ABFE calculations are expected to be extremely difficult or nearly impossible without extraordinarily long simulations or new algorithmic developments. For example, a protein may undergo a substantial and slow conformational change in ligand binding (e.g., HIV-1 protease, which has a large flap motion on inhibitor binding), or a protein may undergo a change in the protonation state on ligand binding (e.g., BACE1, here). In such cases, a normal ABFE calculation, which simply removes the ligand from the binding site, would leave the protein in a metastable unbound state unless simulations are extremely long (in the case of conformational change) or special algorithms are employed (in the case of protonation state changes). In more detail, the two catalytic aspartates in BACE1 were both ionized in the protein–ligand-bound state, but that is likely to change to one ionized and one neutral Asp in the apo state. Thus, we deliberately avoid testing ABFE calculations on BACE1 as we wish to avoid problems caused by an incorrect protonation state for the unbound state. Likewise, we also do not compare to standard RBFE calculations for the scaffold-hopping transformations considered here, as these are simply disallowed by standard RBFE calculations, making comparison impossible. In general, we expect SepTop to be computationally more efficient than ABFE and slightly less efficient than standard RBFE (on transformations where RBFE is applicable).

### 6.4 Molecular Shape and Chemical Similarity May Be Good Metrics for Planning SepTop Calculations

RBFE calculations, including with SepTop, can be planned more effectively using some measure of ligand “similarity” to assess which transformations will be easy and which will be hard. Here, we have not investigated planning and similarity metrics, reserving this for future work. However, it seems likely that SepTop will benefit from different similarity metrics than standard RBFE since SepTop replaces one ligand with another in the binding site rather than mutating one ligand into the other. In this work, we did not explore the impact of the design of the transformation map on the efficiency of the calculations, but we propose that molecular shape and chemical similarity may be good metrics to use for planning SepTop calculation networks, in contrast to the maximum common substructure and 2D graph similarity metrics that are often used in standard RBFE approaches.^12,71^ There is a better chance that ligands introduce similar conformational changes in the protein and that they displace similar water molecules (and therefore converge faster) if the two ligands have a high molecular shape and chemical similarity.

### 6.5 Current Limitations of SepTop

In theory, transformations performed using the SepTop approach are not restricted by the structural similarity of the ligands and ligand binding modes; however, there might be limits to the domain of applicability of the method. We expect slow convergence and biased results if the two ligands bind to different conformations of the protein and if transformations between those conformations happen on timescales longer than the simulation run time. Slow convergence can also be expected if one ligand displaces buried water molecules that are not displaced in the presence of the other ligand, especially if the entry/exit of that water molecule is not sampled throughout the simulation. In addition, results are expected to be incorrect if the ligands bind to different protonation states of the protein or if one or more ligands bind covalently, and results may be slow to converge if the ligands bind in disparate regions of the binding site.

Overall, we found that SepTop performed well on pharmaceutically relevant test systems, which had been previously studied, and then we applied it to a larger number of compounds with two different targets (MALT1 and BACE1) involving both transformations, which are possible for typical RBFE calculations, and those which are challenging or impractical. We found that SepTop performed as well as standard RBFE calculations for transformations within a given congeneric series but avoided complexities of atom mapping and required minimal human intervention to set up the calculations. For scaffold-hopping transformations, accuracy was predictably somewhat lower, likely because these transformations are dramatically more difficult to sample, but these preliminary results are nevertheless encouraging. This suggests that SepTop may be a general and broadly useful approach for RBFE calculations that expand their domain of applicability.

## Supporting Information Available

We provide details on the free energy calculations and 2D structures and experimental affinities for all ligands in this study in Figures S1–S7 and Tables S1–S7 (PDF)
GROMACS topology and coordinate files and parameter files provided as a compressed archive file along with example scripts used in the analysis (MDP files); more detailed explanations (README.md file) (ZIP)
