# Supplementary material for: Broadening the Scope of Binding Free Energy Calculations Using a Separated Topologies Approach
Source: J Chem Theory Comput. 2023 Jul 24;19(15):5058–76. doi: 10.1021/acs.jctc.3c00282 (PMC10413862; doi:10.1021/acs.jctc.3c00282)
Supplement: Supplementary file 1 — ct3c00282_si_001.pdf [file ct3c00282_si_001.pdf]

Supporting Information for Publication: Broadening the scope of binding free energy calculations using a Separated Topologies approach

Hannah M. Baumann,[†] Eric Dybeck,[‡] Christopher Lee McClendon,[‡] Frank C. Pickard IV,[‡] Vytautas Gapsys,[¶] Laura Pérez-Benito,[¶] David F. Hahn,[¶] Gary Tresadern,[¶] Alan M. Mathiowetz,[‡] and David L. Mobley[*,†,§]

†*Department of Pharmaceutical Sciences, University of California, Irvine, CA 92697, United States*

‡*Pfizer Worldwide Research, Development, and Medical, 1 Portland St, Cambridge, MA 02139, United States*

¶*Computational Chemistry, Janssen Research & Development, Janssen Pharmaceutica N. V., Turnhoutseweg 30, B-2340 Beerse, Belgium*

§*Department of Chemistry, University of California, Irvine, CA 92697, United States*

E-mail: dmobley@mobleylab.org

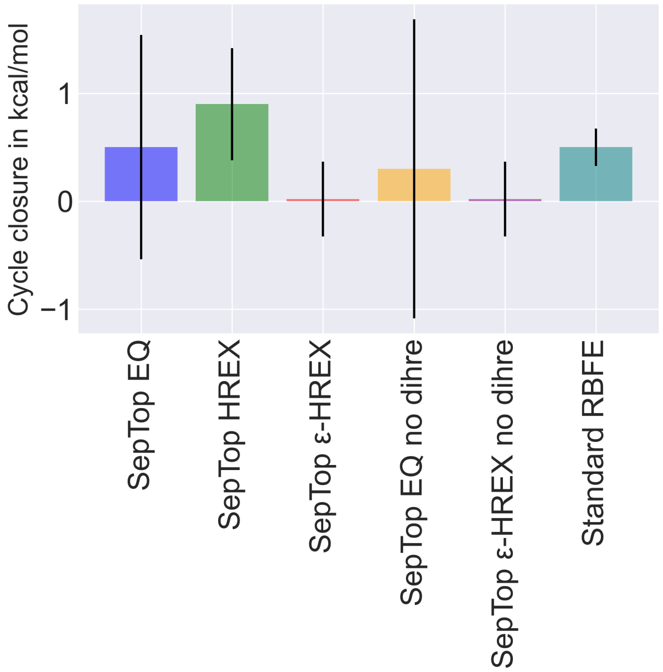

Figure S1. Cycle closure of different alchemical protocols in the ligand cycle in the TYK2 system. The cycle closure was calculated by summing up the $\Delta\Delta G$ values of the three edges of the TYK2 ligand cycle and the absolute values of the cycle closure for five different SepTop protocols and one standard RBFE protocol are shown as bars. The cycle closure was lowest for the two protocols that used ϵ-HREX to enhance sampling, either in combination with restraints on rotatable bonds or without, suggesting that for this system the ϵ-HREX protocols led to good convergence.

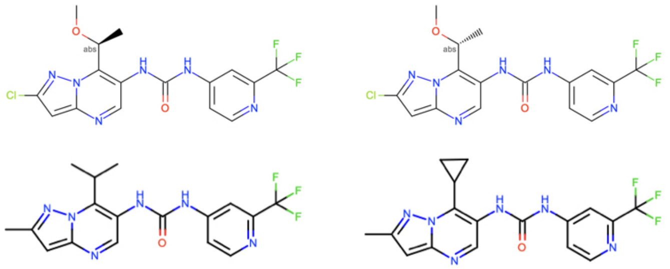

Figure S2. 2D structures of four MALT1 ligands. Transformations here involve a chiral inversion (top) as well as the closing of a ring going from isopropyl to cyclopropyl (bottom).

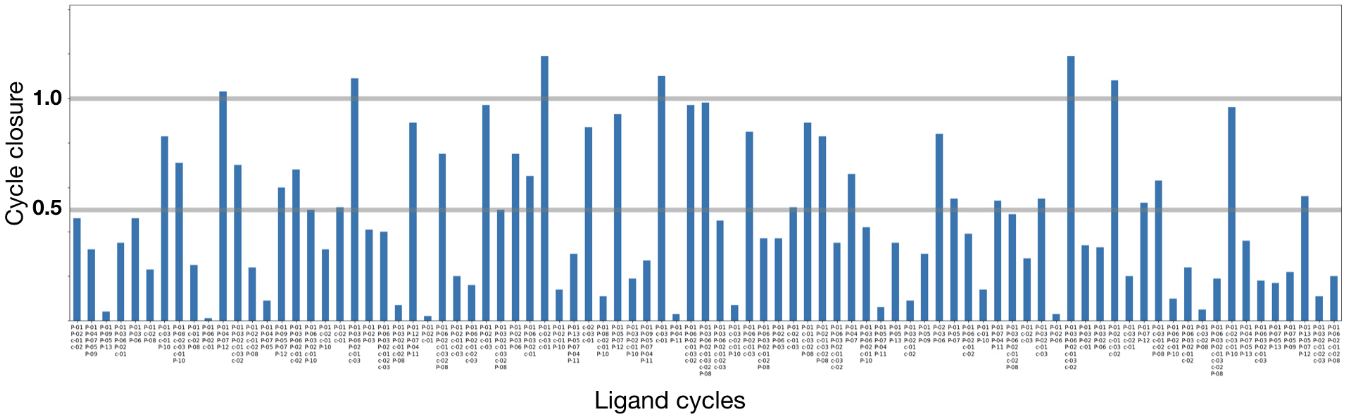

Figure S3. Cycle closure for all ligand cycles in the MALT1 system. The majority of ligand cycles have a cycle closure below 0.5 kcal/mol. Six ligand cycles have a cycle closure greater than 1 kcal/mol, indicating sampling problems.

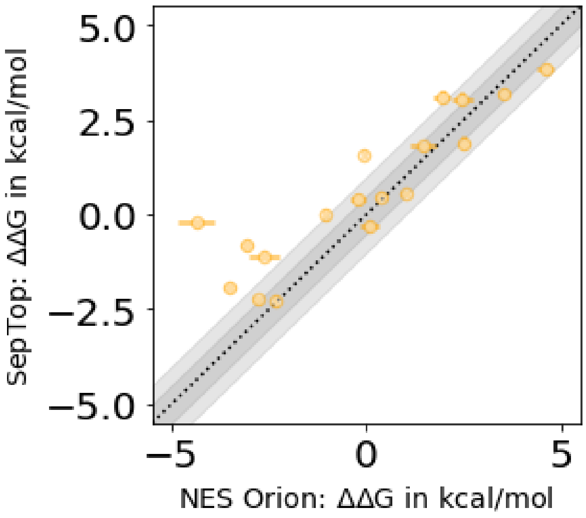

Figure S4. Correlation between SepTop and NES Orion results for the MALT1 system. Shown are ΔΔG values for all edges. For most edges, both methods gave similar results, however, there are some outliers where the two methods do not agree with one another.

Edge	ΔΔG Exp	ΔΔG SepTop	ΔΔG NES
Pfizer-01-01 → Pfizer-01-02	0.26	0.00 ± 0.16	-1.03 ± 0.10
Pfizer-01-01 → Pfizer-01-03	0.52	0.41 ± 0.15	-0.21 ± 0.19
Pfizer-01-02 → Pfizer-01-03	0.26	-0.30 ± 0.16	0.09 ± 0.24
Pfizer-01-01 → Pfizer-01-06	2.19	3.10 ± 0.17	1.94 ± 0.21
Pfizer-01-02 → Pfizer-01-06	1.93	3.05 ± 0.17	2.46 ± 0.27
Pfizer-01-03 → Pfizer-01-06	1.68	1.90 ± 0.16	2.49 ± 0.15
Pfizer-01-01 → compound-01	0.07	-2.24 ± 0.14	-2.78 ± 0.12
Pfizer-01-01 → compound-03	0.46	-0.81 ± 0.14	-3.08 ± 0.16
Pfizer-01-01 → compound-02	-1.18	-1.92 ± 0.14	-3.53 ± 0.14
Pfizer-01-01 → Pfizer-01-04	1.65	3.18 ± 0.14	3.53 ± 0.16
Pfizer-01-01 → Pfizer-01-05	2.16	3.86 ± 0.14	4.61 ± 0.22
Pfizer-01-01 → Pfizer-01-07	2.37	1.81 ± 0.14	1.47 ± 0.36
compound-02 → compound-03	1.64	1.59 ± 0.12	-0.04 ± 0.07
Pfizer-01-05 → Pfizer-01-07	0.21	-1.09 ± 0.13	-2.63 ± 0.40
Pfizer-01-04 → Pfizer-01-07	0.72	-0.22 ± 0.12	-4.37 ± 0.45
compound-02 → compound-01	1.25	0.56 ± 0.12	1.01 ± 0.13
compound-03 → compound-01	-0.38	0.47 ± 0.12	0.38 ± 0.16
Pfizer-01-02 → compound-01	-0.19	-2.28 ± 0.14	-2.35 ± 0.08

Figure S5. Comparing SepTop and NES Orion results in the MALT1 system.

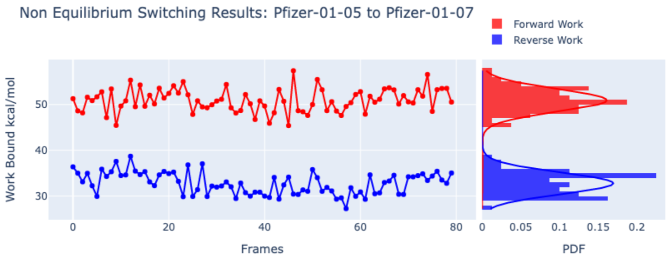

Figure S6. Nonequilibrium work values for the transformation between Pfizer-01-05 and Pfizer-01-07 in the MALT1 system. Forward work values are shown in red and work values from the reverse direction in blue. The distributions of forward and reverse work values do not overlap well for this transformation.

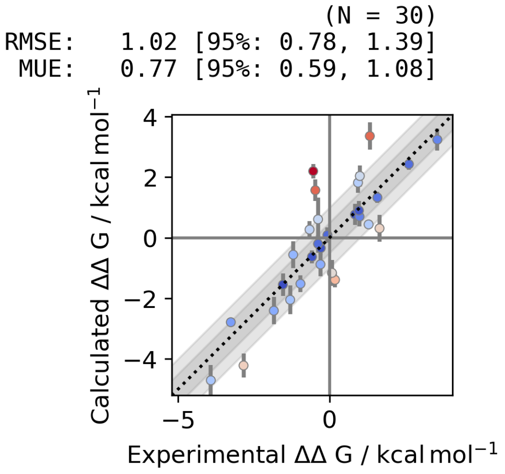

(a)

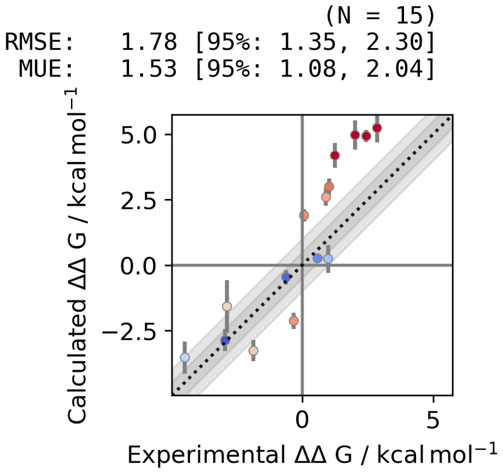

(b)

Figure S7. Correlation between calculated and experimental relative binding free energies for transformations in the BACE1 system. Shown are results from transformations run within the three ligand scaffold series (a) and across different scaffolds (b). $\Delta\Delta$G values from transformations between ligands within the same scaffold (RMSE=1.02) correlate better with experiment than transformations between ligands of different scaffolds (RMSE=1.78).

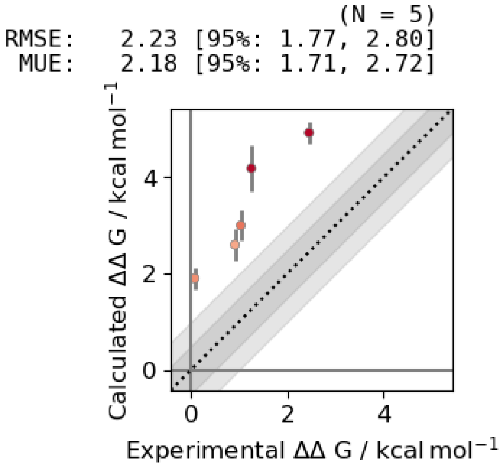

(a) BACE amide biayl

Figure S8. Correlation between calculated and experimental relative binding free energies for transformations between the amide series and the biaryl series in the BACE1 system. For all five transformations the free energy change was calculated to be more unfavorable as to compared to experiment.

Table S1. Relative binding free energies in the ERα system using different input structures. All values are given in kcal/mol. Predictions differed by over 3 kcal/mol depending on the input structure used in the calculations with the most significant difference being in Edge 2d-2e where different input structures led to a difference of 3.7 kcal/mol.

	**Edge 2d - 2e**	**Edge 2d - 3b**	**Edge 2e - 3b**
SepTop (input Spruce)	0.0 ± 0.3	0.4 ± 0.3	0.3 ± 0.3
SepTop (input Aux)	-1.8 ± 0.2	0.9 ± 0.5	3.0 ± 0.4
SepTop (input ATM)	-3.7 ± 0.3	-3.5 ± 0.2	0.3 ± 0.2
ATM [1]	-2.3 ± 0.4	-0.4 ± 0.5	2.1 ± 0.4
Aux [2]		1.3 ± 0.4	2.9 ± 0.5

Table S2. Ligand structures and experimental binding affinities[3] for the TYK2 system.

name	2D structure	Exp. IC50
lig_ejm_42	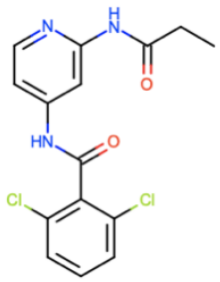	64 nM[3]
lig_ejm_54	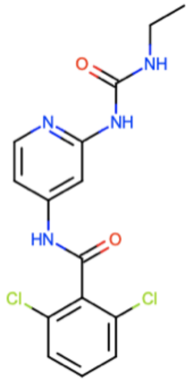	18 nM[3]
lig_ejm_55	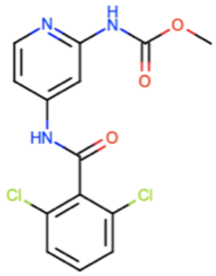	170 nM[3]

Table S3. Ligand structures and experimental binding affinities[4] for the ERα system.

name	2D structure	Exp. IC50
2d	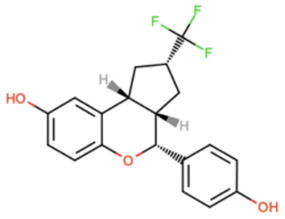	12.4 +/- 4.9 nM[4]
2e	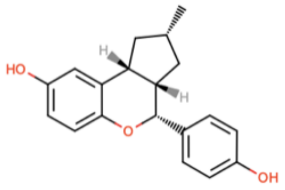	3.3 +/- 2.4 nM[4]
3b	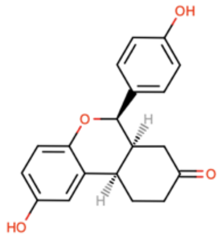	410 nM[4]

Table S4. Ligand structures and experimental binding affinities for the MALT1 system.

name	2D structure	Exp. IC50
2714550-01-1 (compound 1)	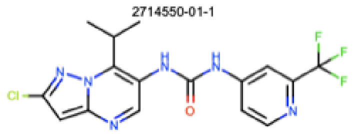	407.4587 nM
1832576-04-1 (compound 2)	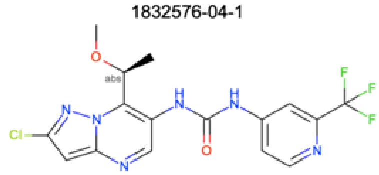	49.257 nM
1832577-09-9 (compound 3)	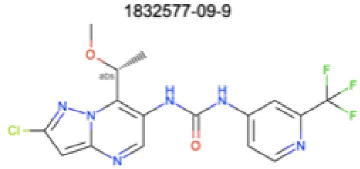	778.82434 nM
Pfizer-01-01	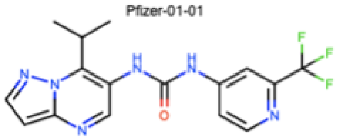	359.95273 nM
Pfizer-01-02	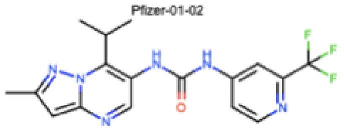	557.5762 nM
Pfizer-01-03	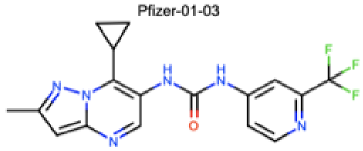	863.8893 nM
Pfizer-01-04	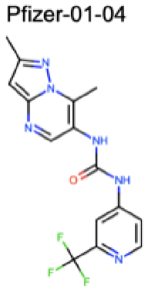	5810.657 nM
Pfizer-01-05	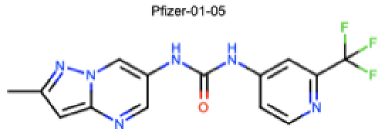	13802.098 nM

Pfizer-01-06	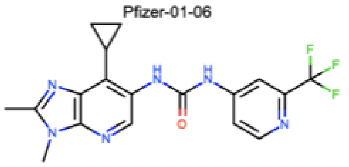	14603.069 nM
Pfizer-01-07	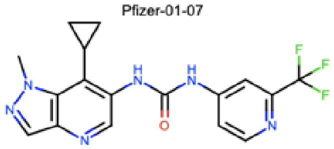	19565.105 nM
Pfizer-01-08	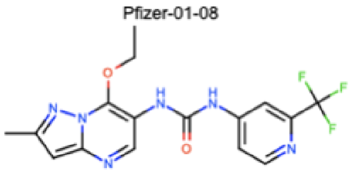	>20000.0 nM
Pfizer-01-09	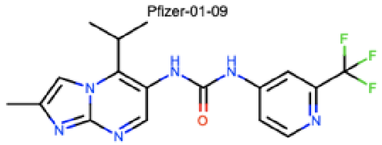	>20000.0 nM
Pfizer-01-10	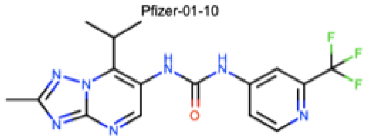	>20000.0 nM
Pfizer-01-11	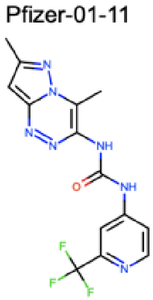	>20000.0 nM
Pfizer-01-12	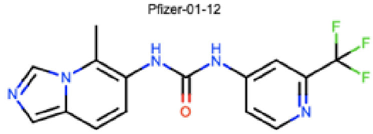	>20000.0 nM
Pfizer-01-13	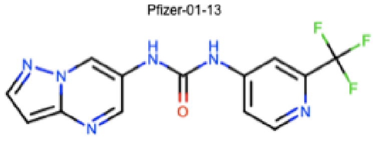	>20000.0 nM

Table S5. Ligand structures and experimental binding affinities[5–9] for the biaryl ligands in the BACE1 system.

name	2D structure	Exp. IC50
lig_02	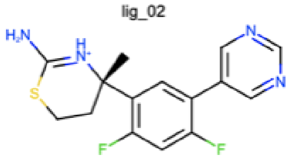	240 nM[5]
lig_03	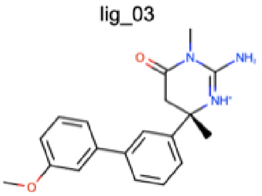	269 nM[6]
lig_04	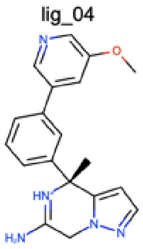	110 nM[7]
lig_05	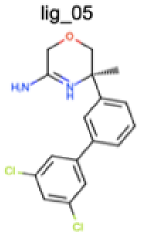	1122 nM[8]
lig_06	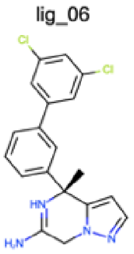	148 nM[7]
lig_07	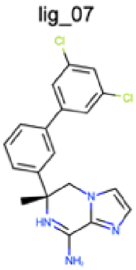	2138 nM[9]

Table S6. Ligand structures and experimental binding affinities[10] for the spirocyclic ligands in the BACE1 system.

lig_1a	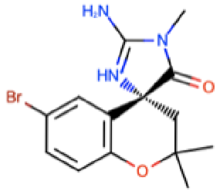	36830 nM[10]
lig_11	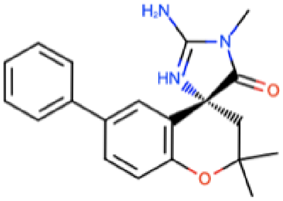	455 nM[10]
lig_13	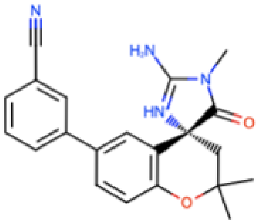	94 nM[10]
lig_16	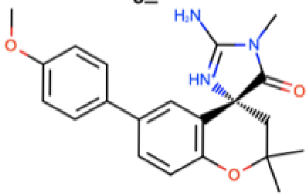	7197 nM[10]
lig_32	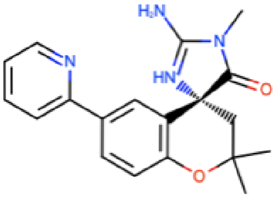	2363 nM[10]
lig_36	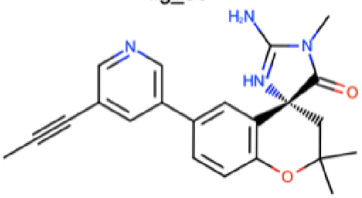	10 nM[10]

Table S7. Ligand structures and experimental binding affinities[11–13] for the amide ligands in the BACE1 system.

lig_41	lig_41 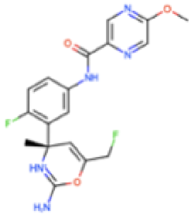	240 nM[11]
lig_45	lig_45 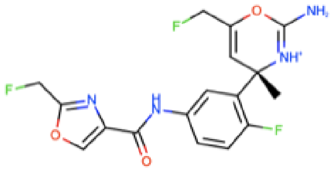	48 nM[11]
lig_67	lig_67 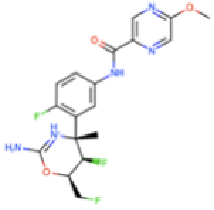	18 nM[12]
lig_69	lig_69 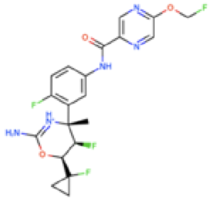	20 nM[12]
lig_74	lig_74 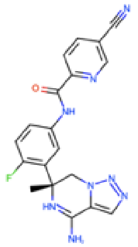	2 nM[13]
lig_81	lig_81 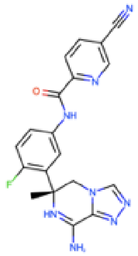	11 nM[13]

References

(1) Azimi, S.; Khuttan, S.; Wu, J. Z.; Pal, R. K.; Gallicchio, E. Relative Binding Free Energy Calculations for Ligands with Diverse Scaffolds with the Alchemical Transfer Method. *Journal of Chemical Information and Modeling* **2022**, *62*, 309–323.

(2) Zou, J.; Li, Z.; Liu, S.; Peng, C.; Fang, D.; Wan, X.; Lin, Z.; Lee, T.-S.; Raleigh, D. P.; Yang, M.; Simmerling, C. Scaffold Hopping Transformations Using Auxiliary Restraints for Calculating Accurate Relative Binding Free Energies. *Journal of Chemical Theory and Computation* **2021**, *17*, 3710–3726.

(3) Liang, J. et al. Lead identification of novel and selective TYK2 inhibitors. *European Journal of Medicinal Chemistry* **2013**, *67*, 175–187.

(4) Richardson, T. I.; Dodge, J. A.; Durst, G. L.; Pfeifer, L. A.; Shah, J.; Wang, Y.; Durbin, J. D.; Krishnan, V.; Norman, B. H. Benzopyrans as selective estrogen receptor Beta agonists (SERBAs). Part 3: Synthesis of cyclopentanone and cyclohexanone intermediates for C-ring modification. *Bioorganic & Medicinal Chemistry Letters* **2007**, *17*, 4824–4828.

(5) Oehlrich, D.; Prokopcova, H.; Gijsen, H. J. The evolution of amidine-based brain penetrant BACE1 inhibitors. *Bioorganic & Medicinal Chemistry Letters* **2014**, *24*, 2033–2045.

(6) Stamford, A. W. et al. Discovery of an Orally Available, Brain Penetrant BACE1 Inhibitor That Affords Robust CNS Aβ Reduction. *ACS Medicinal Chemistry Letters* **2012**, *3*, 897–902.

(7) Trabanco-Suarez, A. A.; Tresadern, G. J.; Delgado-Jimenez, F. 4,7-Dihydropyrazolo[1,5-a]pyrazin-6-yl-amine derivatives as Beta-secretase inhibitors and their preparation and use for the treatment of diseases. *WO2012038438 A1* **2012**,

(8) Trabanco-Suarez, A. A.; Rombouts, F. J. R.; Tresadern, G. J.; Van Gool, M. L. M.; Macdonald, G. J.; Martinez Lamenca, C.; Gijsen, H. J. M. Preparation of 5,6-dihydro-2H-[1,4]oxazin-3-yl-amine derivatives useful as therapeutic inhibitors of Beta-secretase (BACE). *WO2011154431 A1* **2011**,

(9) Trabanco-Suarez, A. A.; Delgado-Jimenez, F.; Vega Ramiro, J. A.; Tresadern, G. J.; Gijsen, H. J. M.; Oehlrich, D. Preparation of 5,6-dihydro-imidazo[1,2-a]pyrazin-8-ylamine derivatives as beta-secretase inhibitors. *WO2012085038 A1* **2012**,

(10) Hunt, K. W. et al. Spirocyclic β-Site Amyloid Precursor Protein Cleaving Enzyme 1 (BACE1) Inhibitors: From Hit to Lowering of Cerebrospinal Fluid (CSF) Amyloid β in a Higher Species. *Journal of Medicinal Chemistry* **2013**, *56*, 3379–3403.

(11) Fuchino, K. et al. Rational Design of Novel 1,3-Oxazine Based Beta-Secretase (BACE1) Inhibitors: Incorporation of a Double Bond To Reduce P-gp Efflux Leading to Robust Abeta Reduction in the Brain. *Journal of Medicinal Chemistry* **2018**, *61*, 5122–5137.

(12) Nakahara, K. et al. Discovery of Potent and Centrally Active 6-Substituted 5-Fluoro-1,3-dihydro-oxazine Beta-Secretase (BACE1) Inhibitors via Active Conformation Stabilization. *Journal of Medicinal Chemistry* **2018**, *61*, 5525–5546.

(13) Oehlrich, D. et al. Evaluation of a Series of Beta-Secretase 1 Inhibitors Containing Novel Heteroaryl-Fused-Piperazine Amidine Warheads. *ACS Medicinal Chemistry Letters* **2019**, *10*, 1159–1165.
